# Tillering plasticity of drought-stressed barley genotypes under different re-watering regimes

**DOI:** 10.1186/s12870-025-07504-8

**Published:** 2025-10-13

**Authors:** Sahel Soleimani, Philip Wichmann, Klaus Pillen, Kerstin Neumann, Andreas Maurer

**Affiliations:** 1 Institute of Agricultural and Nutritional Sciences, Martin Luther University Halle-Wittenberg, Betty-Heimann-Str. 3, 06120 Halle, Germany; 2 Leibniz Institute of Plant Genetics and Crop Plant Research (IPK), 06466 Seeland, Germany

**Keywords:** Barley (Hordeum vulgare ssp. vulgare), Wild barley (Hordeum vulgare ssp. spontaneum), Re-watering, Re-tillering, Tiller number, Drought stress

## Abstract

**Background:**

One future challenge of agriculture will be maintaining food security in times of climate change. Future plant breeding, therefore, has to account for the genotypes’ survival during drought and a good recovery ability after rainfall events. We aimed at investigating the re-tillering behavior of selected barley genotypes (cultivars and wild barley introgression lines) in different drought and re-watering scenarios, which were applied in a high-throughput phenotyping facility. Re-tillering describes the activation of additional tillers after post-stress irrigation.

**Results:**

Twenty-three selected genotypes of the barley NAM population HEB-25, along with three control genotypes, were evaluated in four replicates under five distinct treatment conditions. In this experiment, re-tillering was particularly pronounced in an alternating watering and stress treatment, where the resumption of irrigation post-stress enabled full recovery of the plants. However, it was noted that while re-watering after a stress period promotes tiller activation and the development of fertile ears, it also tends to increase the number of sterile ears. The degree of sterile ear formation varied significantly among different genotypes, highlighting the critical role of genetic factors in modulating plant responses to re-tillering and post-stress irrigation. It is important to note that excessive re-tillering has been shown to exhibit a negative correlation with fertile ear weight.

**Conclusions:**

The impact of focusing on tiller number and re-tillering behavior in future barley breeding may be significant, particularly in the context of climate change. By selecting for genotypes with appropriate tillering plasticity, breeders can develop barley varieties that are more resilient to stress conditions such as drought.

**Supplementary Information:**

The online version contains supplementary material available at 10.1186/s12870-025-07504-8.

## Background

Barley (*Hordeum vulgare L*.) is one of the oldest and most significant cereal crops cultivated by humans [1]. Domestication of barley took place approximately 10,500 years ago [2]. Barley is a globally significant cereal crop renowned for its adaptability to diverse environmental conditions and its economic importance in agriculture, food, brewing and distilling industries [3]. Barley yield stability is far better than of other cereals, making it a dependable source of food in stress-prone seasons [4].

The global climate is predicted to change drastically over the next century and various parameters will be affected in this changing environment [5]. Generally, different climate models concertedly predict that precipitation events will decrease in quantity but increase in intensity. This will lead to more extended periods of drought, followed by periods of heavy rainfall. Drought stress poses a formidable challenge to barley production worldwide, affecting crop growth, development, and ultimately yield [6]. During drought conditions, reduced water availability disrupts various physiological processes in plants, including tillering, which is crucial for determining final grain yield [7].

Tillers are the lateral branches that grow from the main shoot or basal meristem of non-elongated internodes and produce their adventitious roots and spikes during their development [8]. Tillering, one of the major agronomic traits in cereal crops, is closely related to grain yield formation and stability [9]. Further, tillering is also influenced by many environmental factors, including water [10], nutrients [11], temperature [12] and light [13].

The response of barley genotypes to drought stress and subsequent re-watering regimes varies widely, influencing tiller initiation, development, and survival [14]. Different genotypes exhibit varying degrees of resilience in terms of tiller number and subsequent recovery of grain yield upon rehydration [15].

Understanding the tillering dynamics of drought-stressed barley under different re-watering regimes is essential for developing strategies to mitigate the adverse effects of water scarcity on crop productivity [14]. The tillering dynamics of barley has been studied extensively from the 1960 s until the 1990 s, resulting in the general assumption that the maximum tiller number can be observed around the beginning of stem extension, followed by partial tiller reduction during stem extension until a stabilization from anthesis to harvest occurs [16]. However, several studies observed deviations from this general rule, as certain genotypes show late tillering even after anthesis under certain natural environments [17] and in controlled experiments [18–21]. This re-tillering often occurs after rainfall events [22] succeeding periods of early drought [18, 20] scenario that becomes more and more tangible as the weather extremes increase in times of climate change.

The objective of this research is to investigate the re-tillering behavior of selected barley genotypes (cultivars and wild barley introgression lines) in different drought and re-watering scenarios, which were applied in a high-throughput automated plant phenotyping platform for medium-sized plants (APPP-B at IPK Gatersleben, Germany) to identify the genotypes that exhibit greater productive recovery ability after re-watering. For this, the tillering dynamics of 26 diverse genotypes was determined over time, distinguishing between fertile (productive) and sterile (non-productive) tillers at harvest.

## Methods

### Plant material

The nested association mapping (NAM) population ‘Halle Exotic Barley’ (HEB-25) consists of 25 families with 1,420 lines in BC_1_S_3_ generation, resulting from initial crosses between the spring barley elite cultivar Barke (*Hordeum vulgare ssp. vulgare*) and 25 highly divergent exotic barley accessions (*Hordeum vulgare ssp. spontaneum* and *ssp. agriocrithon)* [23]. In total, 23 HEB-25 lines were selected for this study. The selection was based on the tillering behavior in previous experiments on the same platform under well-watered and drought-stress conditions, as well as grain yield performance in field trials. Lines were chosen to represent contrasting tillering under stress and control conditions, based on their absolute and relative differences, as well as high grain yield under control and field conditions. For details about their selection, see Additional file 1. Additionally, three control cultivars were included: ‘Barke’ (the reference parent of HEB-25), ‘RGT Planet’ (a high-yielding malting barley cultivar with impressive environmental plasticity [24], bred by RAGT Seeds) and ‘Keel’ (an Australian feed barley variety often used in drought stress experiments and chosen for its contrasting agronomic traits).

### Trial setup

**Fig. 1:**
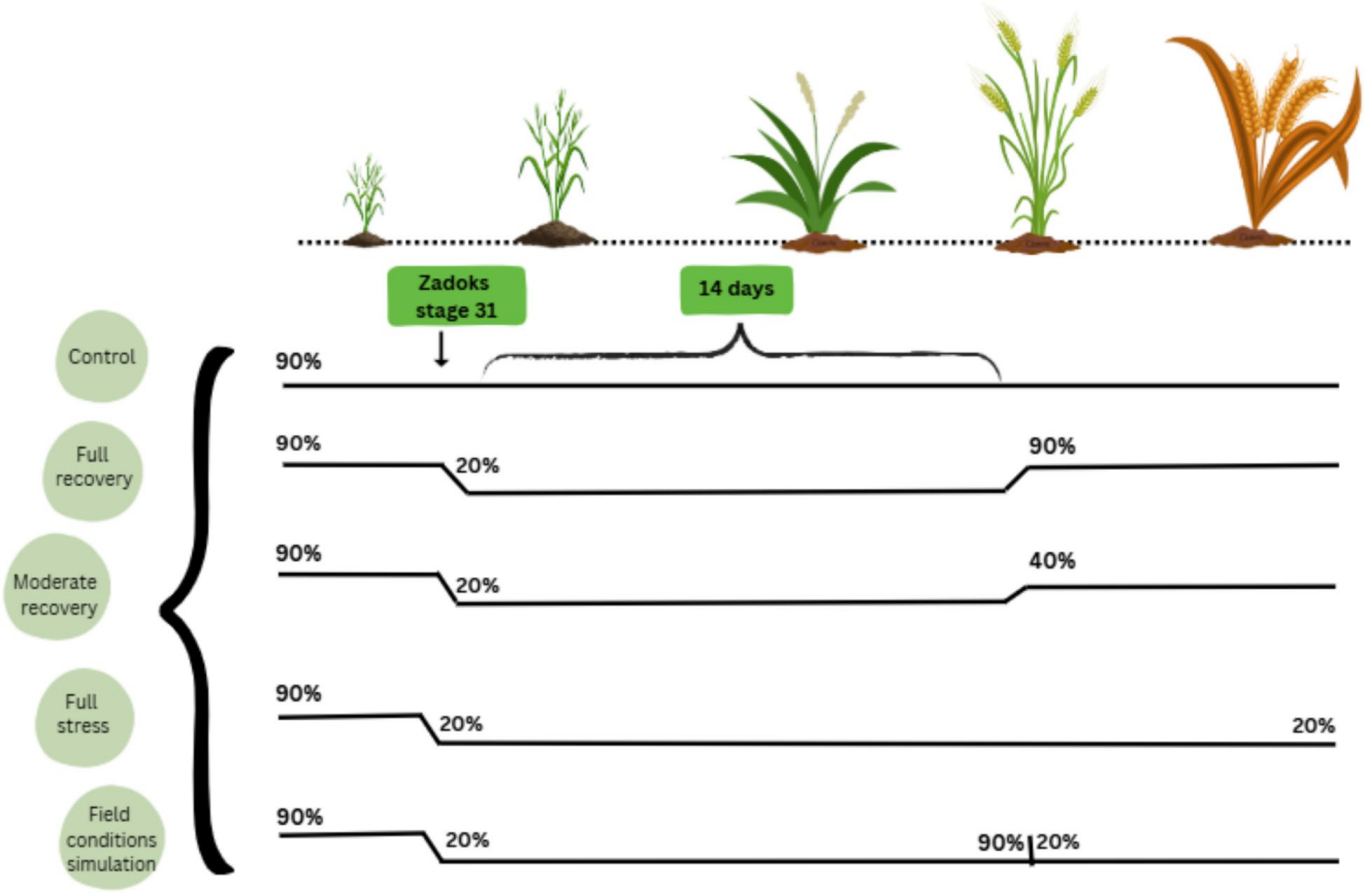
Treatments and their different watering regimes. This figure illustrates the different treatments and their different watering targets based on field capacity (FC). The treatments include Control (90% FC), Full recovery (90–20–90% FC), Moderate recovery (90–20–40% FC), Full stress (90–20-20% FC) and Field conditions simulation (90–20–90–20% FC). Each horizontal line represents the watering target per treatment during specific time intervals. Figure created using *Canva* [26]

We used the non-invasive automated plant phenotyping platform for medium-sized plants (APPP-B) of the Leibniz Institute of Plant Genetics and Crop Plant Research (IPK) in Gatersleben, Germany. This system includes a conveyor belt enabling daily RGB imaging (side and top view of the shoot) and automated watering of 520 pots [25]. Each pot was automatically weighed before and after irrigation, allowing precise determination of water uptake and enabling controlled watering to predefined soil water content levels. The images were collected for documentation purposes only; no traits were extracted from them. We investigated the phenomenon of re-tillering and its potential impact under five different watering regimes, simulating various drought stress scenarios. We germinated and transplanted the lines into pots (capacity of 2 L, height of 19.5 cm and top diameter of 14.5 cm) containing established soil mixtures as reported in [25]. The total of 26 genotypes was replicated 4 times per treatment (i.e., watering regime), resulting in a total of 520 pots (Additional file 2). Each pot contained a single plant. The genotypes were grown under a 16-h day/8-h night regime with temperatures of 20 °C during the day and 18 °C at night. For each pot, the different drought and re-watering scenarios were programmed individually, ensuring that the daily watering based on the intended field capacity was maintained. Watering was initially set to a target amount corresponding to 90% of field capacity. Each individual plant was screened for its growth stage three times a week until stem elongation was reached to initiate the watering regime according to Fig. 1 and Additional file 3.

For the control treatment (90%) watering was set to 90% of field capacity (FC) throughout the entire experiment. For the full recovery treatment (90–20–90%) watering was set to 90% FC until Zadoks stage 31 (Z31) [27]. After reaching this stage the pots were not watered until 20% FC was reached, which took 15–18 days. This level was then maintained for 14 days. Then, the pots were re-watered to 90% FC for the rest of the experiment. The same watering strategy was applied in the moderate recovery treatment (90–20–40% FC) with the only difference that plants were re-watered to 40% instead of 90 FC until the end of the experiment. For the full stress treatment (90–20-20% FC), plants were not re-watered but maintained at 20% FC until the end of the experiment. In the field conditions simulation treatment (90–20–90–20% FC) plants were re-watered to 90% FC only once and then dried down again to maintain a field capacity of 20% until maturity (Fig. 1). After 2 months, when all respective plants had received the targeted re-watering, they were transferred to a greenhouse to grow until maturity. This was necessary to avoid long plants falling from the conveyor belt during movement. The greenhouse conditions were maintained as indicated above (16-h day/8-h night regime with temperatures of 20 °C during the day and 18 °C at night), and the different watering regimes were continued manually (Additional file 4).

### Phenotyping of traits

Tiller number was manually determined from four days after transplanting (DAT) and continued until 57 DAT, with assessments conducted at seven different time points (4, 9, 16, 21, 30, 37 and 57 DAT). Measurements were intended to be conducted once a week, while the last measurement was taken prior to transferring the plants to the greenhouse. Re-tillering was defined as the difference in tiller number at 57 DAT between the Full recovery (90–20–90%) and the Full stress (90–20-20%) treatments. This definition was based on the assumption that the 90–20-20% treatment can be seen as the reference of no re-tillering, while we expected to see most re-tillering in the 90–20–90% treatment, receiving the largest water amount upon re-watering. Upon reaching maturity, plants were harvested. During this process, the final plant height was measured and recorded in centimeters (cm) from the ground to the end of the longest shoot including the ear. Subsequently, the shoot parts of the plants were cut and weighed. The number of ears per plant was manually counted. Ears were categorized into fertile and sterile ears (with less than 50% seed set), each of which was counted and weighed separately. Consequently, the term fertile ear weight represents the sum of all fertile ears, which is the total grain yield. Harvest index was calculated by dividing the grain yield by the total biomass. After threshing, seeds were cleaned and remaining awns were manually removed to avoid contamination in the analysis of grain parameters. The cleaned grains were then utilized to determine the thousand-grain weight and grain number using the seed analyzer MARViN (MARViTECH GmbH, Wittenburg, Germany).

### Statistical analysis

Phenotypic data were analyzed using RStudio [28]. To investigate the distribution of traits across different treatments and genotypes, box plots were generated with the R package ggplot2 [29]. These visualizations helped to compare the spread, central tendency and to identify potential outliers for each trait. An ANOVA including fixed main effects (treatment and genotype) was conducted to check for significant genotype × treatment (G × T) effects for each trait using the `aov` function in R [28]. Following ANOVA, a Tukey’s Honest Significant Difference (HSD) test was performed to identify specific group differences using the `TukeyHSD` function in R [28]. To evaluate the correlation between traits within each treatment group the `cor` function in R [28] was used based on trait means per genotype. To calculate the slope of the tiller number across the different treatments for each genotype the `dplyr ` package in R [28] was used for data grouping, while the slope was calculated using the `lm` and ` coef ` functions from base R. Additionally, the average fertile ear weight of each treatment was correlated with grain yield data of six different field environments based on 22 shared genotypes [30]. Pearson correlation coefficients were calculated to assess the strength and direction of linear relationships in both analyses.

In addition to the ANOVA and correlation analyses, repeatability of the traits was assessed using the `rptR` package in RStudio [31]. This analysis was conducted to evaluate the consistency of trait measurements across different conditions or time points, providing insights into the reliability of the observed data. 

## Results

### Analysis of variance

The examined quantitative traits (plant height, shoot weight, tiller number (57 DAT), ear number, fertile ear number, sterile ear number, fertile ear weight, sterile ear weight, grain number, thousand grain weight) were evaluated by ANOVA to study the effects of genotype and treatments (Table 1). Genotype, treatments and the genotype × treatment interaction influenced all traits significantly. Specifically, p-values for genotype, treatment, and the genotype × treatment interaction were all below the conventional significance level of 0.001, confirming that these factors have a significant effect on the traits examined. The significant effect of genotype indicates that there are inherent differences among genotypes concerning trait expression. The significant treatment effect suggests that the applied treatments had a differential impact on the traits. The significant genotype × treatment interaction reveals that the response to treatment varies depending on the genotype, highlighting the need for a tailored approach in applying treatments based on genotype. All traits exhibited significant variability across treatments (Table 2). The control treatment yielded the highest mean values for plant height, shoot weight, ear number, fertile ear number, fertile ear weight, and grain number. In contrast, the mean tiller number was greater in the full recovery (90–20–90%) treatment relative to other treatments, indicating the occurrence of re-tillering in this setup. Compared to the control treatment, this increase in tiller number suggests that these plants were able to produce new shoots after experiencing a period of stress, known as re-tillering. Furthermore, the mean values for sterile ear number and sterile ear weight were also elevated in the full recovery treatment, suggesting a notable proportion of ears failed to produce viable grains. The highest mean thousand grain weight was recorded under the 90–20–90–20% treatment (field conditions simulation), indicating that this water regime promoted grain development compared to the other treatments (Table 2).

**Table 1:** Analysis of variance (ANOVA) for traits

Source of variation	F value	*P* value
Plant_height		
Genotype	56.859	<.001
Treatment	440.837	<.001
Genotype × Treatment	3.144	<.001
Shoot_weight		
Genotype	93.516	<.001
Treatment	749.512	<.001
Genotype × Treatment	3.519	<.001
Ear_number		
Genotype	17.530	<.001
Treatment	245.071	<.001
Genotype × Treatment	3.286	<.001
Tiller_number (57 DAT)		
Genotype	27.908	<.001
Treatment	192.883	<.001
Genotype × Treatment	2.481	<.001
Fertile_ear_number		
Genotype	17.17	<.001
Treatment	280.99	<.001
Genotype × Treatment	2.45	<.001
Sterile_ear_number		
Genotype	10.92	<.001
Treatment	27.664	<.001
Genotype × Treatment	3.26	<.001
Fertile_ear_weight		
Genotype	19.27	<.001
Treatment	280.583	<.001
Genotype × Treatment	2.362	<.001
Sterile_ear_weight		
Genotype	7.815	<.001
Treatment	29.144	<.001
Genotype × Treatment	2.350	<.001
Grain_number		
Genotype	15.154	<.001
Treatment	225.927	<.001
Genotype × Treatment	2.124	<.001
Thousand_grain_weight		
Genotype	17.237	<.001
Treatment	58.301	<.001
Genotype × Treatment	2.244	<.001

**Table 2:** Descriptive statistics for traits

Trait	Treatment	Mean	Tukey group	Min	Max	SD	CV(%)	Repeatability
Plant Height [cm]	90%	100.80	a	41	138	15.94	15.82	0.84
	90–20–90%	91.89	b	36	124	15.31	16.66	0.74
	90–20–40%	82.66	c	41	106	10.94	13.24	0.72
	90–20-20%	70.61	d	35	88	8.90	12.61	0.62
	90–20–90–20%	72.86	d	35	99	9.86	13.54	0.73
Shoot Weight [g]	90%	27.52	a	1.70	41.10	7.69	27.95	0.88
	90–20–90%	20.81	b	1	33.50	6.95	33.39	0.80
	90–20–40%	16.52	c	1.60	26.40	4.92	29.81	0.84
	90–20-20%	11.71	d	1.20	19.90	4.06	34.67	0.74
	90–20–90–20%	13.43	d	1.10	24.40	4.30	32.07	0.83
Tiller Number (57 DAT)	90%	32.94	a	12	59	8.71	26.47	0.72
	90–20–90%	34.82	a	0	68	10.90	31.30	0.69
	90–20–40%	26.13	b	7	47	7.18	27.48	0.56
	90–20-20%	18.01	d	8	34	6.90	38.60	0.54
	90–20–90–20%	22.33	c	7	61	7.43	33.28	0.45
Ear Number	90%	27.20	a	6	51	7.30	26.86	0.67
	90–20–90%	21.54	b	0	45	8.21	38.10	0.46
	90–20–40%	19.80	b	3	39	5.72	28.87	0.46
	90–20-20%	11.84	c	1	27	4.49	37.98	0.53
	90–20–90–20%	11.77	c	2	32	5.98	50.82	0.68
Fertile Ear Number	90%	23.53	a	4	38	6.70	28.51	0.50
	90–20–90%	15.23	b	0	35	6.88	45.21	0.39
	90–20–40%	14.61	b	2	28	4.84	33.14	0.65
	90–20-20%	9.32	c	1	23	3.48	37.39	0.65
	90–20–90–20%	8.19	c	1	20	3.66	44.72	0.69
Sterile Ear Number	90%	3.67	bc	0	23	5.20	141.76	0.54
	90–20–90%	6.52	a	0	29	5.25	80.58	0.51
	90–20–40%	5.19	ab	0	18	3.98	76.71	0.41
	90–20-20%	2.51	c	0	10	2.55	101.38	0.22
	90–20–90–20%	3.58	c	0	15	3.44	96.18	0.51
Fertile Ear Weight [g]	90%	23.54	a	1	46.4	9.46	40.19	0.52
	90–20–90%	10.52	bc	0	26.6	6.03	57.27	0.49
	90–20–40%	11.86	b	0.9	20.6	4.23	35.67	0.57
	90–20-20%	8.30	cd	0.4	17.1	3.41	41.14	0.71
	90–20–90–20%	6.96	d	1.3	13.9	3.15	45.26	0.80
Sterile Ear Weight [g]	90%	0.70	bc	0	5.2	1.06	151.57	0.44
	90–20–90%	1.19	a	0	7.1	1.12	93.90	0.30
	90–20–40%	0.82	b	0	2.9	0.66	80.23	0.38
	90–20-20%	0.35	d	0	2.3	0.41	115.06	0.36
	90–20–90–20%	0.43	cd	0	1.6	0.40	94.04	0.55
Grain Number	90%	353.30	a	4	688	157.71	44.64	0.43
	90–20–90%	146.75	bc	0	404	95.53	65.09	0.40
	90–20–40%	176.53	b	4	321	76.10	43.11	0.60
	90–20-20%	117.86	cd	13	236	54.70	46.41	0.66
	90–20–90–20%	98.79	d	10	239	50.15	50.76	0.74
Thousand Grain Weight [g]	90%	42.32	c	26.96	58.84	6.96	16.45	0.44
	90–20–90%	38.56	d	27.47	51.33	5.08	13.19	0.50
	90–20–40%	43.32	bc	26.25	56.09	5.71	13.20	0.61
	90–20-20%	45.62	ab	27.30	59.09	5.57	2.22	0.63
	90–20–90–20%	46.38	a	29.11	58.48	5.83	12.58	0.44

### Tillering dynamics analysis

**Fig. 2:**
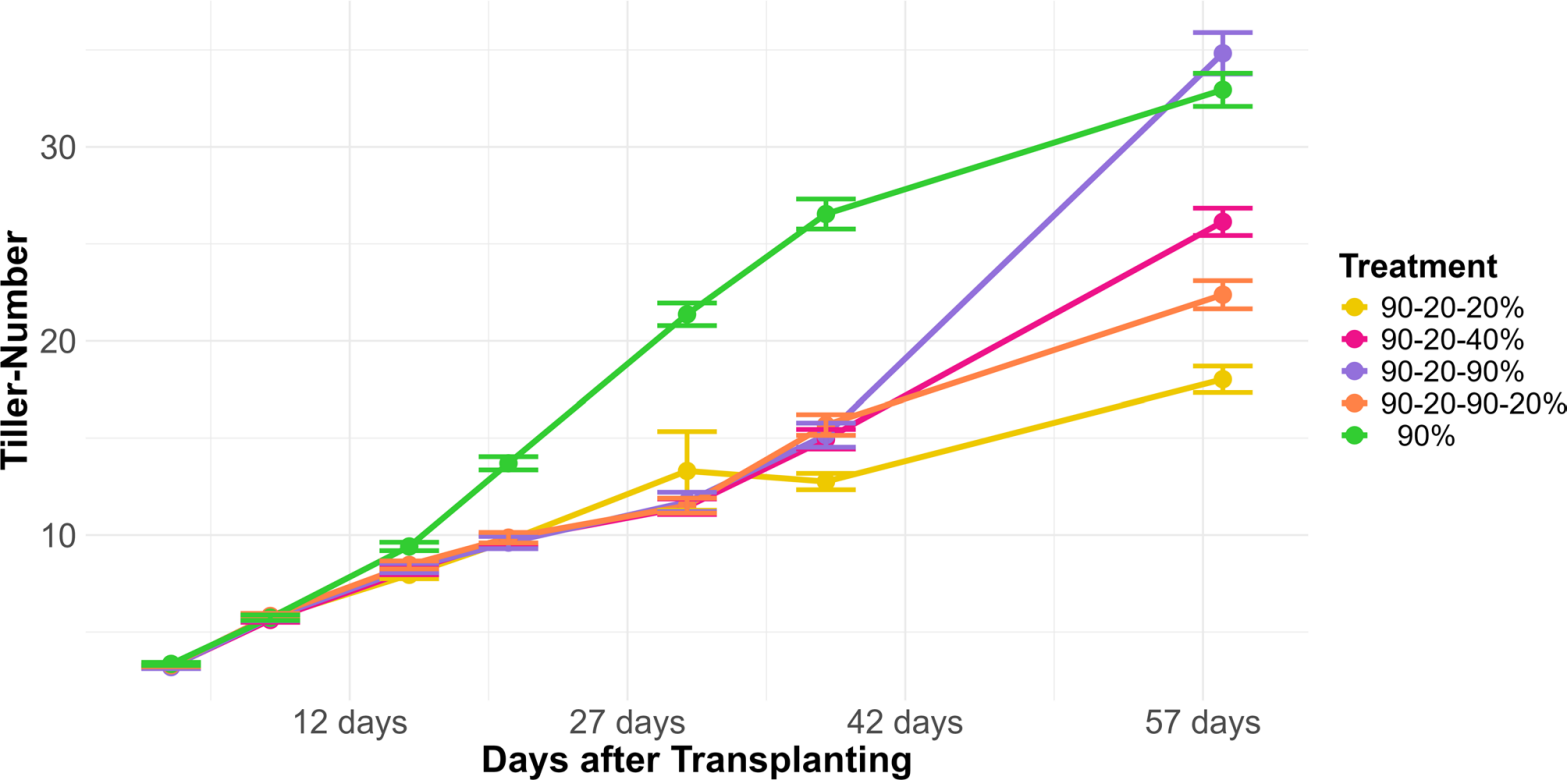
Tiller count at various temporal intervals. This plot depicts the mean number of tillers, including the standard error, observed at different days after transplanting across all genotypes. Each treatment is represented by a distinct line color, illustrating the changes in tiller count during the experiment. The stress period for each plant varied but was approximately from 17 days after transplanting to 32 days after transplanting. Each dot on the plot represents a specific time point where tiller counts were recorded, providing a temporal snapshot of the tiller dynamics under different treatment conditions

Tiller counting is an essential practice in agriculture for evaluating the growth and yield potential of cereal crops such as barley. We initiated tiller counting at 4 DAT and continued until 57 DAT, with assessments conducted at 7 different time points. The different treatments showed varying responses in re-tillering following re-watering (Fig. 2).

**Fig. 3:**
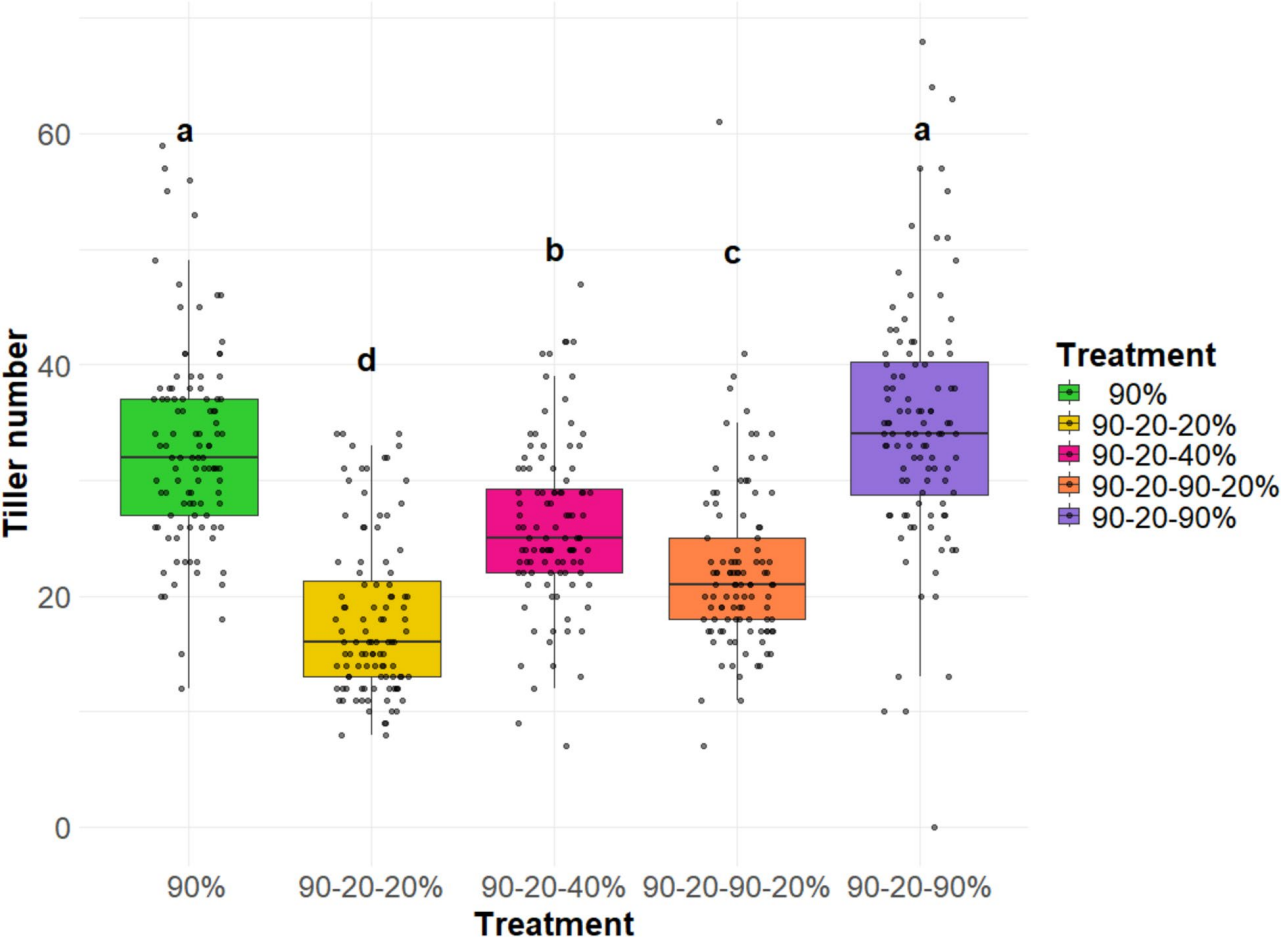
Tiller number for five watering treatments at 57 DAT. Each box-and-whisker plot represents the distribution of the data of 26 genotypes in 4 replications. The lower and upper edges of the box correspond to the first (Q1) and third quartiles (Q3), respectively, while the median is shown as a horizontal line within the box. The whiskers extend to the smallest and largest values within 1.5 times the interquartile range (IQR = Q3—Q1) from the quartiles. Data points beyond this range are plotted as outliers. The statistical analysis was performed using a one-way ANOVA followed by Tukey’s Honest Significant Difference (HSD) test to assess the differences between treatments. Statistically significant differences (P < 0.05) are marked by different letters

**Fig. 4:**
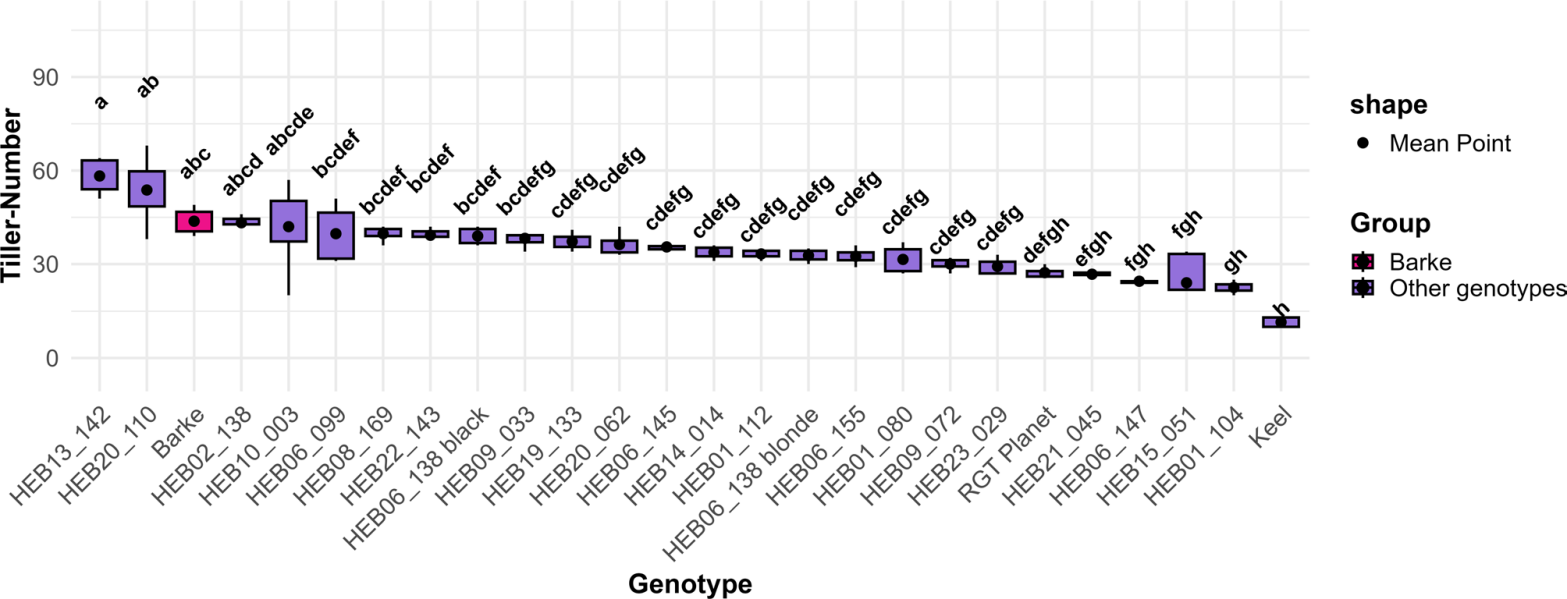
Tiller number at 57 DAT of 26 genotypes under 90–20–90% (full recovery) treatment. Box-and-whisker plots display the distribution of the data of 4 replications, while the mean is shown as a dot. The statistical analysis was performed using a one-way ANOVA followed by Tukey’s Honest Significant Difference (HSD) test to assess the differences between different genotypes. Statistically significant differences (P < 0.05) are marked by different letters

**Fig. 5:**
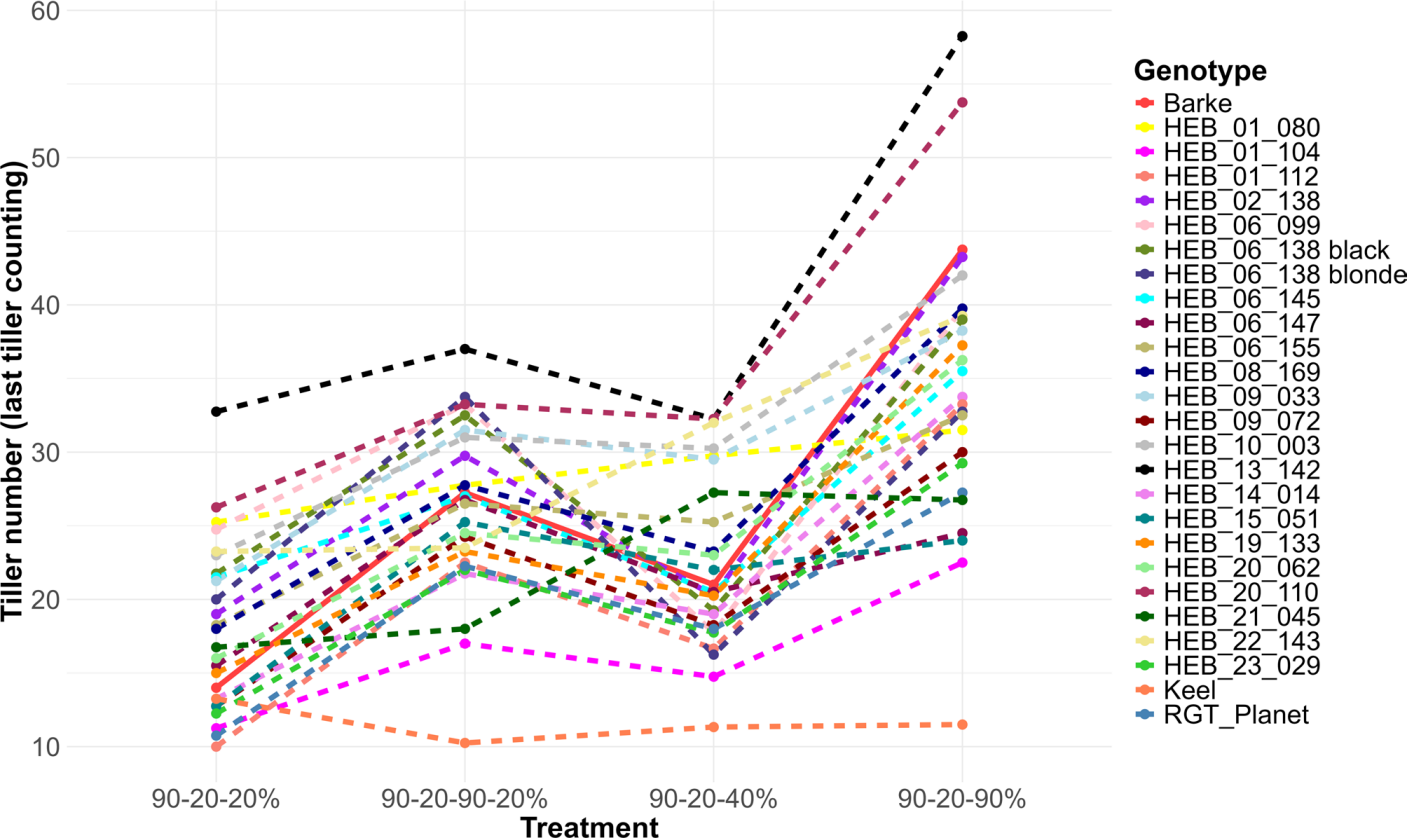
Tiller number at 57 DAT for 26 genotypes under four re-watering treatments. Each genotype is represented by a distinct line color, illustrating the changes in tiller number between treatments. The solid red line represents the genotype Barke, while the dotted lines represent the other genotypes. Genotypes per treatment are indicated as mean values across four replicates

**Fig. 6:**
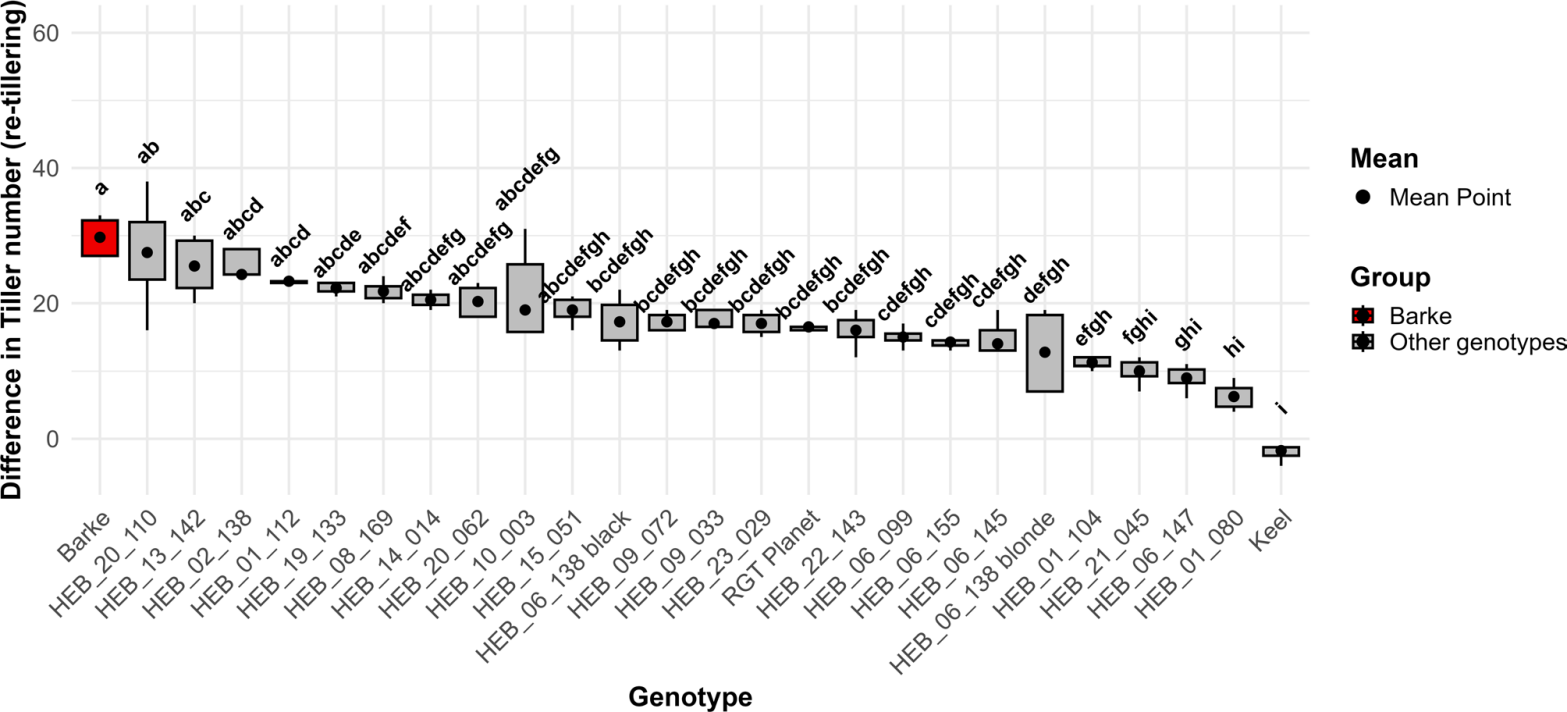
Tiller number difference (re-tillering) between 90–20-20% and 90–20–90% treatments across 26 genotypes. Re-tillering was calculated by determining the difference in tiller numbers between the 90–20–90% (full recovery) and 90–20-20% (full stress) treatments at 57 DAT. Box-and-whisker plots display the distribution of the data of 4 replications, while the mean is shown as a dot. The statistical analysis was performed using a one-way ANOVA followed by Tukey’s Honest Significant Difference (HSD) test to assess the difference between genotypes. Statistically significant differences (P < 0.05) are marked by different letters

Plants subjected to the 90–20–90% (full recovery) treatment exhibited significantly more tillers compared to the other re-watering treatments (Fig. 3). The tiller number was found to increase in line with the quantity of water applied following re-watering, suggesting a response to water supply. Among the 26 genotypes tested, none exhibited a significantly higher tiller number than the control genotype Barke, under the 90–20–90% (full recovery) treatment (Fig. 4), although two HEB lines showed in tendency a higher tiller number at 57 DAT. Contrasting, Keel had significantly the lowest tiller number among all genotypes. The variations in appearance, specifically in plant height and shoot density between plants subjected to different re-watering regimes were easily visible (Additional file 5). We also examined the tillering responses of all genotypes to the re-watering treatments (Fig. 5). Among the genotypes, HEB_20_110 exhibited the highest re-tillering potential, followed by HEB_13_142. In contrast, Keel was one of the genotypes that did not exhibit re-tillering. Accordingly, the re-tillering behavior the difference in tiller numbers between the 90–20-20% (full stress) and 90–20–90% (full recovery) treatments differed among the genotypes, with Barke having the strongest re-tillering capacity and Keel the lowest (Fig. 6).

**Fig. 7:**
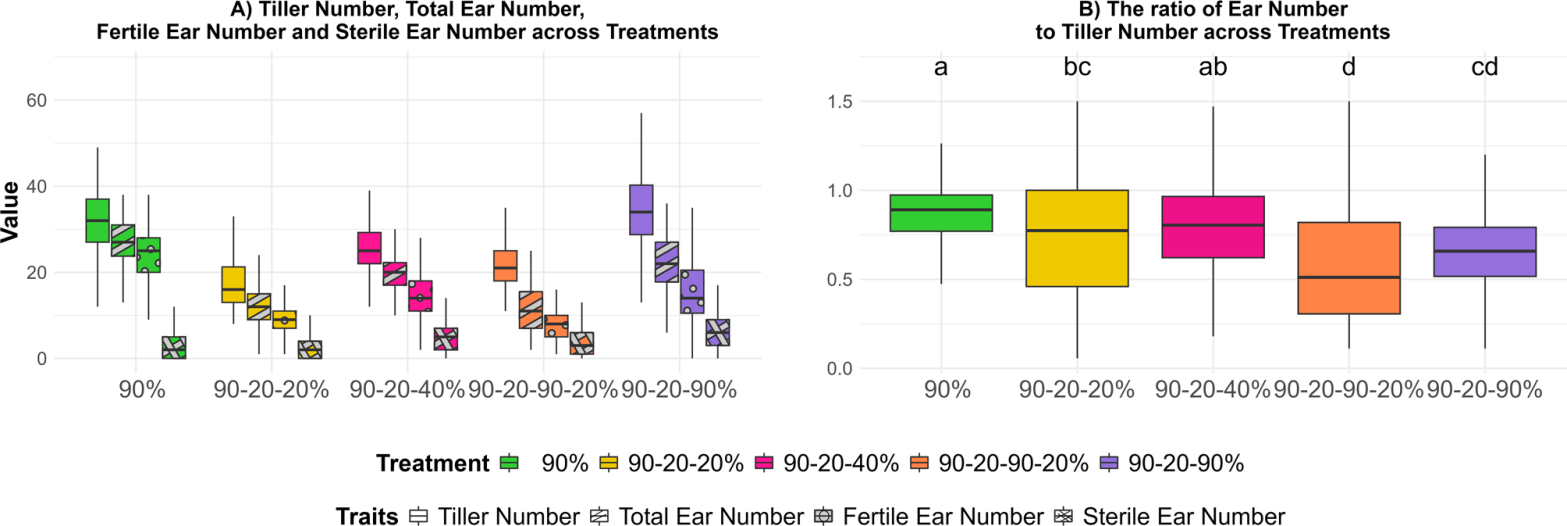
Comparison of tiller and ear numbers for five treatments. The treatments are represented by different colors, while patterns are used to distinguish between traits. Each box-and-whisker plot displays the distribution of the data of 26 genotypes in 4 replications, while the median is shown as a horizontal line. **A** Tiller number, ear number, fertile ear number and sterile ear number for different treatments. **B** Ratio of ear number to tiller number for five treatments. The statistical analysis was performed using a one-way ANOVA followed by Tukey’s Honest Significant Difference (HSD) test to assess the differences between treatments. Statistically significant differences (*P* < 0.05) are marked by different letters. Values can exceed a value of 1, as the tiller number was determined 7 weeks before harvest

The utility of tiller number in plants is contingent upon the successful development of tillers into ears, culminating in fertile ears. As water stress increases, the ability of barley plants to produce tillers is affected. Under optimal conditions, a greater number of tillers can develop, which can subsequently transition to fertile ears. However, during drought stress, the number of tillers that develop and successfully convert to fertile ears decreased in all our drought treatments compared to the control treatment (Fig. 7A). The progression of tillers into ears is influenced by a multitude of factors including genetic predisposition, environmental conditions and agronomic practices. An increased number of tillers does not necessarily translate to a higher number of ears. Figure 7B illustrates the ratio of ear number to tiller number in the various treatments. After the 90% (control) treatment, the 90–20–40% (moderate recovery) treatment exhibited a high percentage of tillers that successfully developed into ears. Conversely, the 90–20–90% (full recovery) treatment, despite having a higher tiller number than the other treatments, demonstrated a low ratio of ear number to tiller number. This indicates that a substantial proportion of tillers did not progress to ear development. Regarding the 90–20–90–20% (field conditions simulation) treatment, the ratio of ear number to tiller number was the lowest.

**Fig. 8:**
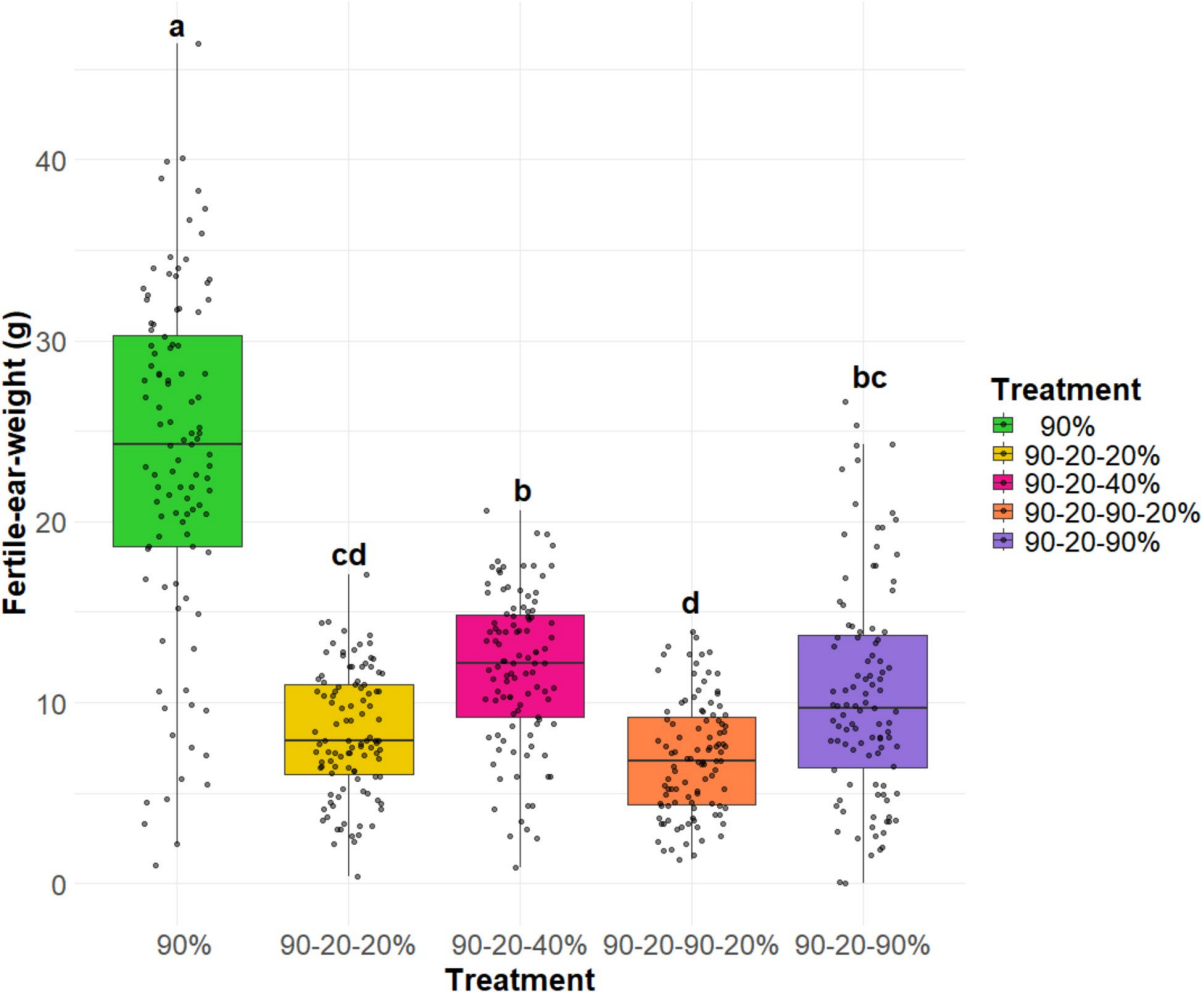
Fertile ear weight (single plant grain yield) for five treatments. Each box-and-whisker plot displays the distribution of the data of 26 genotypes in 4 replications, while the median is shown as a horizontal line. The statistical analysis was performed using a one-way ANOVA followed by Tukey’s Honest Significant Difference (HSD) test to assess the differences between treatments. Statistically significant differences (*P* < 0.05) are marked by different letters. All samples were analyzed in four replicates for 26 genotypes

**Fig. 9:**
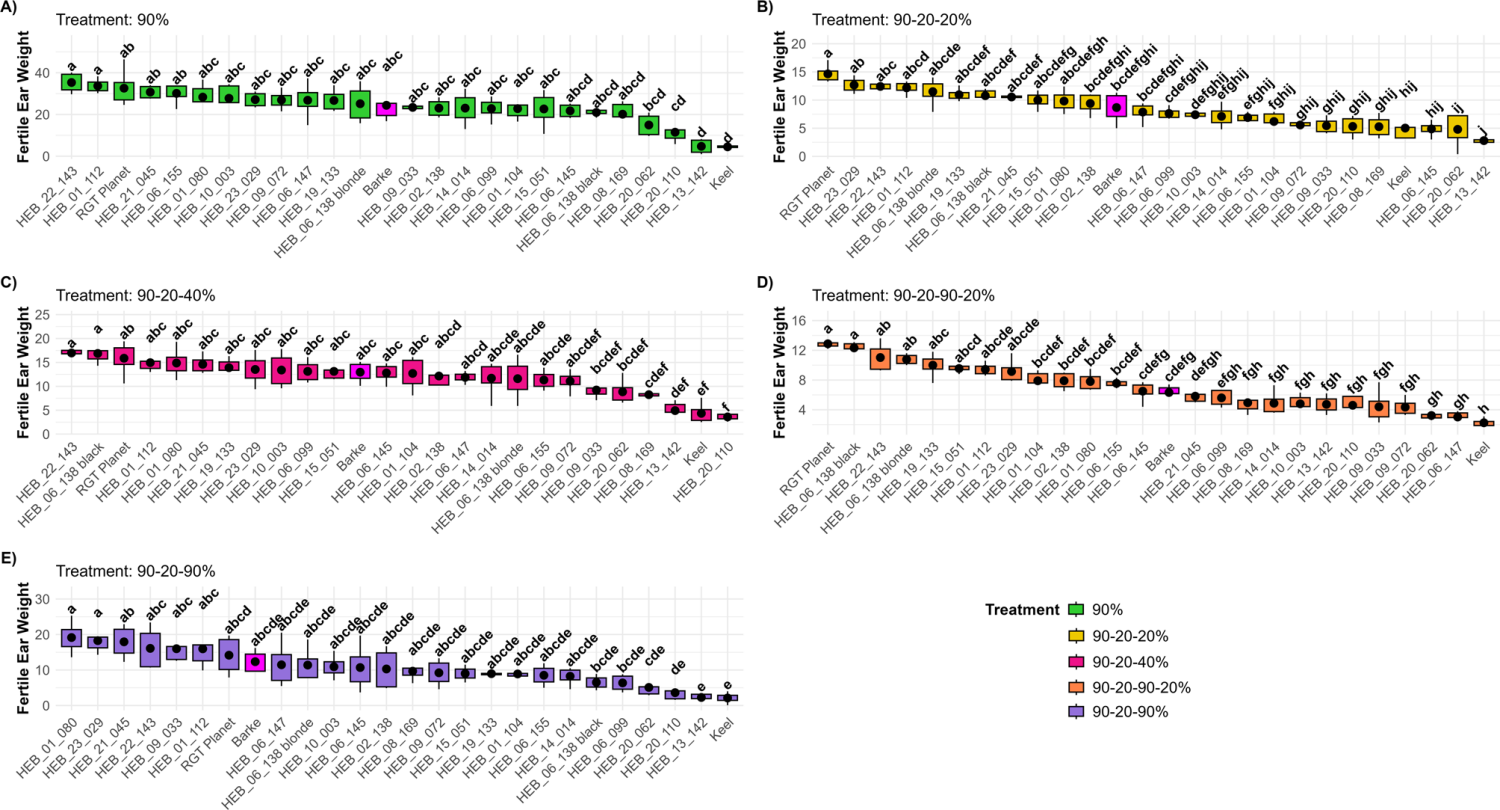
90–20-20% (full stress treatment),Fertile ear weight (g) for 26 genotypes under five treatment conditions. This figure presents the results of five box plots (A-E), each representing the performance of 26 genotypes in one of the five treatment conditions: A) 90% (control treatment), B) 90-20-20% (full stress treatment), C) 90-20-40% (moderate recovery treatment), D) 90-20-90-20% (field conditions simulation treatment), E) 90-20-90% (full recovery treatment). Box-and-whisker plots display the distribution of the data of 4 replications per genotype, while the mean is shown as a dot. Statistical analyses were performed using one-way ANOVA followed by Tukey’s Honest Significant Difference (HSD) test to assess the differences between 26 genotypes. Statistically significant differences (P < 0.05) are marked by different letters. The color coding in the figure represents the treatment groups, where the "Barke" genotype is shown in magenta, and the others are represented in one color (green, purple, pink, yellow, orange) based on the treatment.

**Fig. 10:**
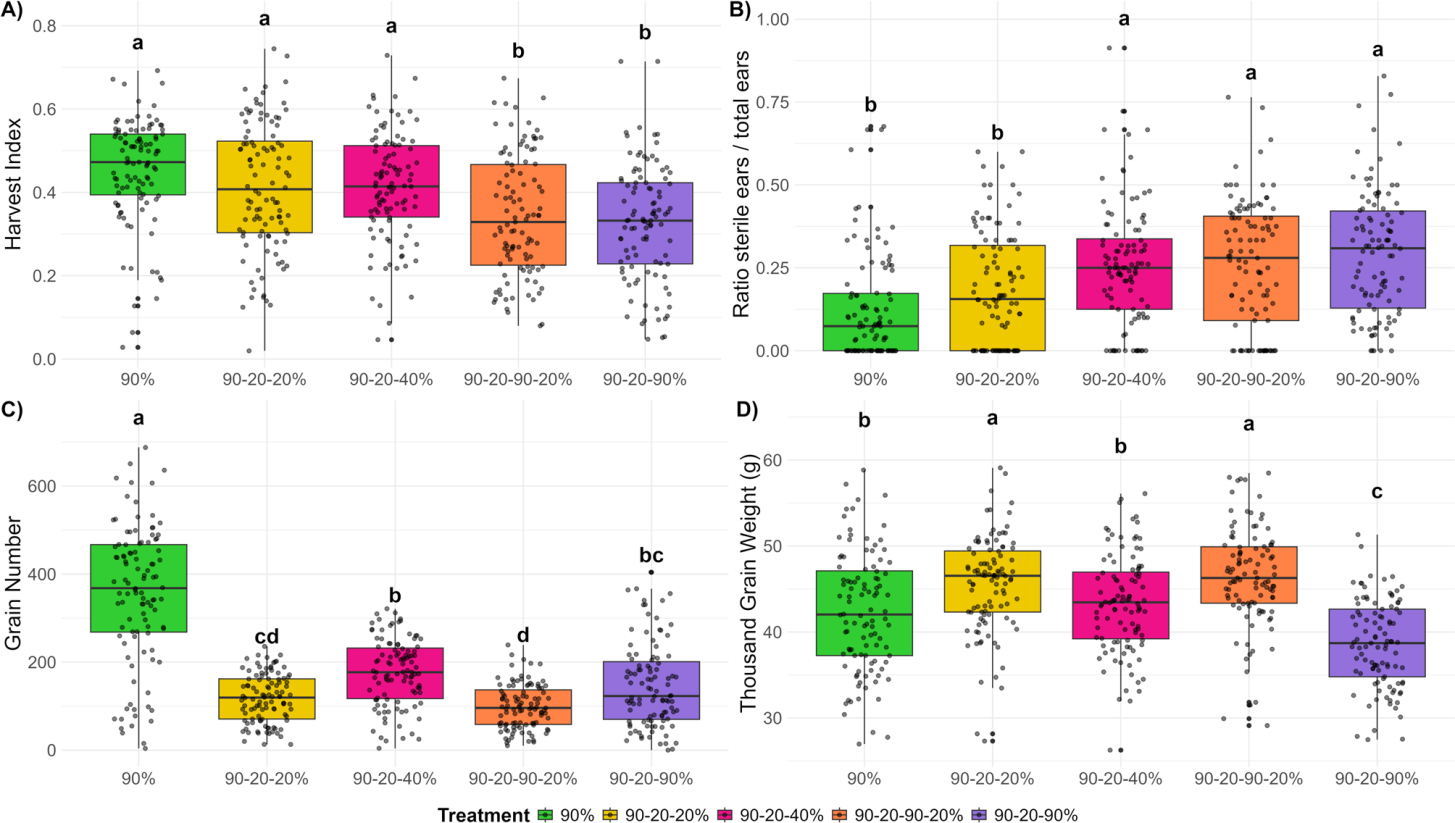
Effects of five treatments on four traits. This figure presents the distribution of harvest index (**A**), ratio of sterile to total ear number (**B**), grain number (**C**) and thousand grain weight (**D**) under five treatments represented by different colors. Each box-and-whisker plot displays the distribution of the data of 26 genotypes in 4 replications, while the median is shown as a horizontal line. The statistical analysis was performed using a one-way ANOVA followed by Tukey’s Honest Significant Difference (HSD) test to assess the differences between treatments. Statistically significant differences (*P* < 0.05) are marked by different letters

Tiller number and growth are essential for determining the overall yield of barley. Thus, evaluating fertile ear weight is essential for assessing the performance of different barley genotypes under various environmental conditions. This trait is used to identify high-yielding and drought resilient genotypes suitable for specific growing conditions. In this research, among all treatments, the 90–20–40% (moderate recovery) treatment exhibited a high fertile ear weight, second only to the 90% (control) treatment (Fig. 8). Conversely, although the 90–20–90% (full recovery) treatment exhibited a higher tiller number compared to the 90–20–40% (moderate recovery) treatment, its fertile ear weight (single plant grain yield) was lower, indicating suboptimal ear and seed development relative to the 90–20–40% (moderate recovery) treatment. The 90–20–90–20% (field conditions simulation) treatment displayed the lowest fertile ear weight (single plant grain yield), even lower than the 90–20-20% (full stress) treatment, despite having a higher tiller number. This suggests that while tiller development was relatively enhanced, ear and grain development remained incomplete in the 90–20–90–20% (field conditions simulation) treatment. Figure 9 presents a box plot illustrating the distribution of fertile ear weight across 26 barley genotypes under various treatments. In the 90% (control) treatment (Fig. 9A), 12 genotypes had a higher mean fertile ear weight than Barke, with HEB_22_143 and HEB_01_112 showing the highest values among the genotypes suggesting a tendency towards higher ear weight in these genotypes. Under the 90–20–90% (full recovery) treatment (Fig. 9B), 7 genotypes exhibited a higher mean fertile ear weight than Barke. Among these, HEB_01_080 and HEB_23_029 had the highest values, while HEB_20_110, HEB_13_142, and Keel were among those with the lowest. For the 90–20–40% (moderate recovery) treatment (Fig. 9C), 11 genotypes displayed a higher mean fertile ear weight than Barke, although these differences were not statistically significant. Among them, HEB_22_143 and HEB_06_138black showed the highest mean fertile ear weights. In the 90–20-20% (full stress) treatment (Fig. 9D), 11 genotypes also surpassed Barke in mean fertile ear weight, with RGT Planet being the only genotype that differed significantly from Barke. Finally, under the 90–20–90–20% (field conditions simulation) treatment (Fig. 9E), 13 genotypes demonstrated a higher mean fertile ear weight than Barke. Among these, RGT Planet, HEB_06_138black, HEB_06_18blonde and HEB_22_143 significantly exceeded Barke. To evaluate the impact of different treatments on plant productivity, we calculated the harvest index for each treatment. The first three treatments (90%, 90–20-20%, and 90–20–40%) showed a higher harvest index and were statistically similar to each other, suggesting that under these conditions, plants allocate resources more efficiently to harvestable biomass (i.e. grain yield). In contrast, the 90–20–90–20% (field conditions simulation) and 90–20–90% (full recovery) treatments resulted in a lower harvest index, indicating less efficient resource allocation towards the grains under those conditions (Fig. 10A).

The ratio of sterile ears to total ears was calculated (Fig. 10 B) and the three re-watering treatments exhibited a significantly higher ratio of sterile ears compared to both the control and full stress treatments. This elevated ratio in the re-watering treatments suggests that the initial water stress, followed by re-watering, adversely affected reproductive development, likely leading to incomplete recovery or irreversible damage to ear fertility.

The total grain number varied across all genotypes and treatments. The control treatment (90%) had significantly more grains than all other treatments as shown in Fig. 10C. Further, the moderate stress treatment (90–20–40%) showed significant differences compared to the full stress (90–20-20%) and field conditions simulation (90–20–90–20%) treatment. Between the Full stress (90–20-20%) and field conditions simulation (90–20–90–20%) treatment were no significant differences in the number of grains observed.

The thousand grain weight varied among all genotypes across different treatments (Fig. 10D). The 90–20–90–20% treatment showed a significantly higher thousand grain weight compared to the control (90%), the 90–20–40% (moderate recovery), and 90–20–90% (full recovery) treatments. As fewer grains were produced under 90–20–90–20% (field conditions simulation) and 90–20-20% (full stress) treatments (Fig. 10C), allowing for greater nutrient distribution to each grain, resulting in a higher carbohydrate concentration per grain.

### Trait correlations

**Fig. 11:**
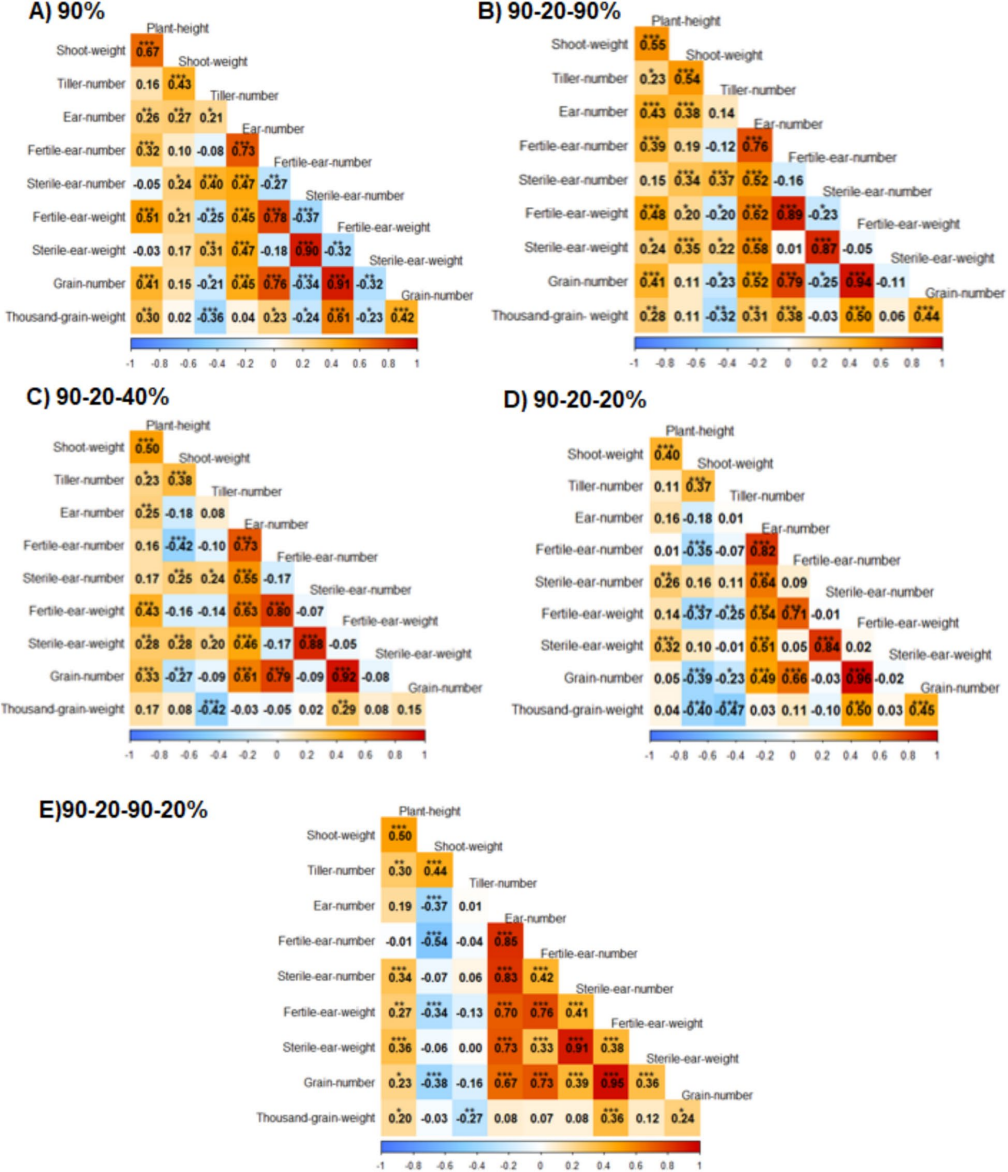
Pearson’s correlation coefficients between 10 examined traits for 90% (control) treatment (**A**), 90–20–90% (full recovery) treatment (**B**), 90–20–40% (moderate recovery) treatment (**C**), 90–20-20% (full stress) treatment (**D**) and 90–20–90–20% (field conditions simulation) treatment (**E**). Significant correlation coefficients are indicated with * *P* < 0.05, **P < 0.01 and *** *P* < 0.001

**Fig. 12:**
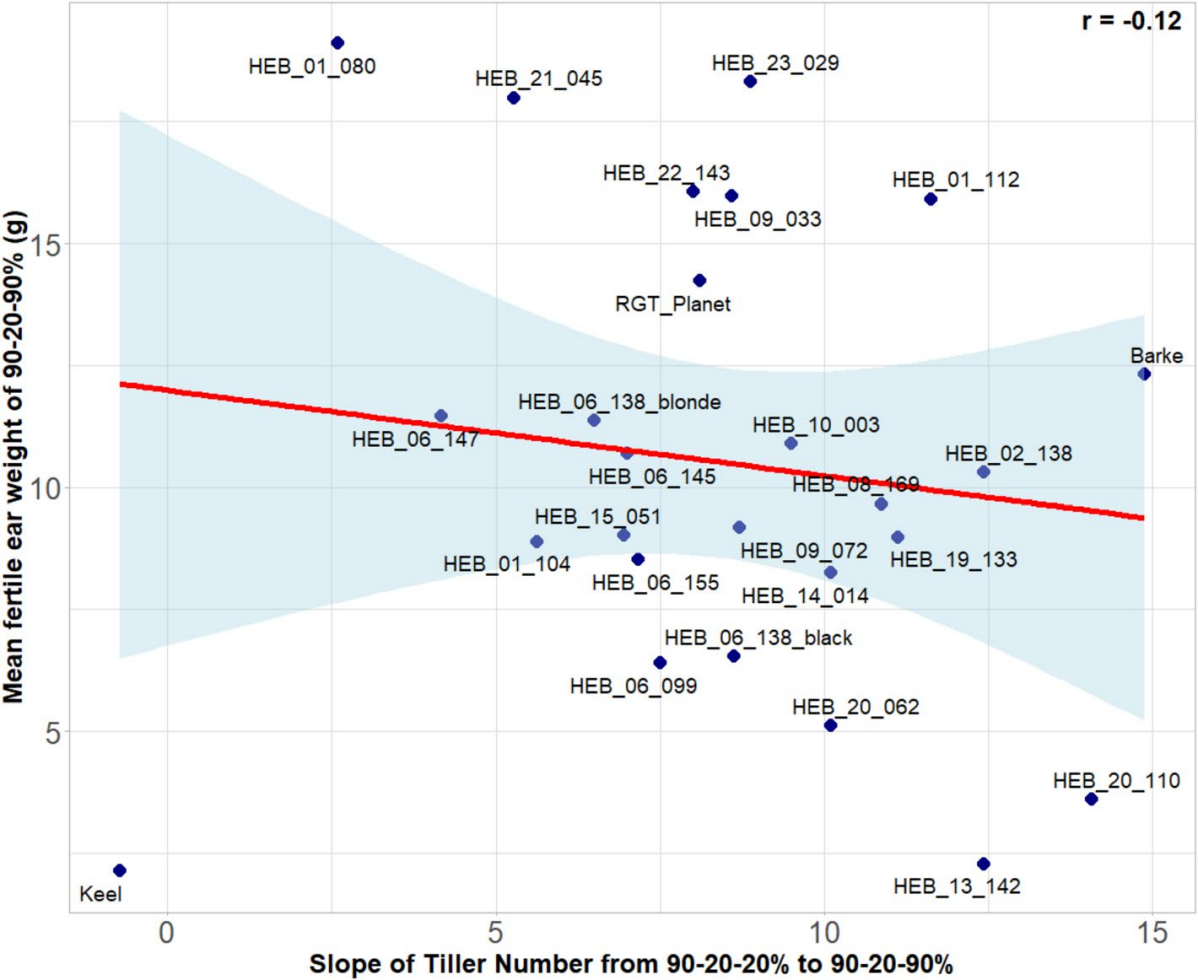
Scatter plot of the slope of tiller number from 90–20-20% (full stress) over 90–20–40% (moderate recovery) to 90–20–90% (full recovery) with the fertile ear weight of the 90–20–90% (full recovery) treatment. The scatter plot displays individual data points, with each dot representing a genotype mean across 4 replicates. The x-axis represents the slope of tiller number and the y-axis shows the mean fertile ear weight of 90–20–90% treatment. A linear regression line illustrates the strength and direction of the relationship. The correlation is clearly biased by the genotype Keel. The Pearson’s correlation coefficient (r) is included to quantify this relationship, revealing the degree of association between slope of tiller number and fertile ear weight

**Fig. 13:**
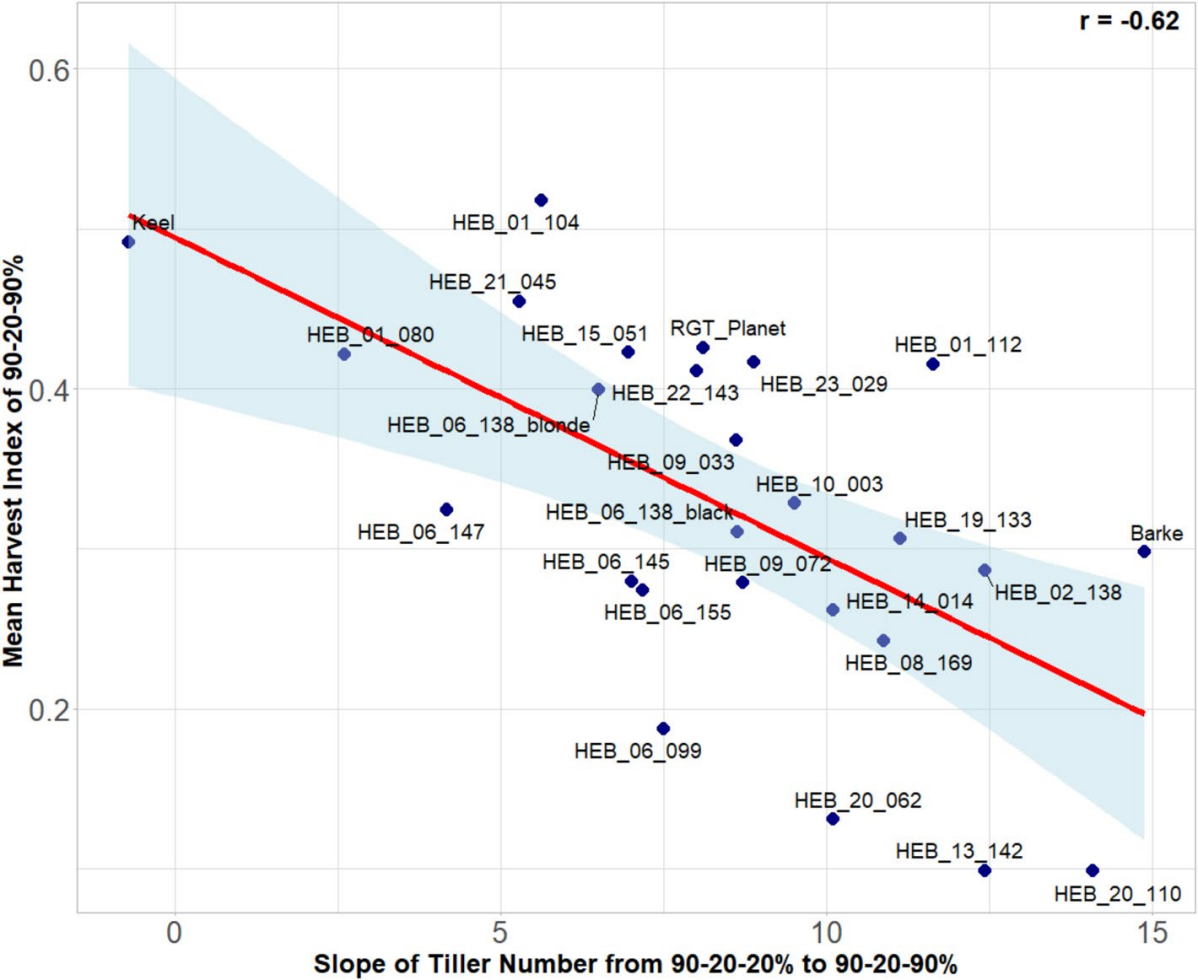
Pearson’s correlation of the slope of tiller number from 90–20-20% (full stress) over 90–20–40% (moderate recovery) to 90–20–90% (full recovery) with the Harvest Index of the 90–20–90% (full recovery) treatment. The scatter plot displays individual data points, with each dot representing a genotype mean across 4 replicates. The x-axis represents the slope of tiller number, and the y-axis shows the mean Harvest Index of 90–20–90% treatment. A linear regression line illustrates the strength and direction of the relationship. The Pearson’s correlation coefficient (r) is included to quantify this relationship, revealing the degree of association between slope of tiller number and Harvest Index

**Fig. 14:**
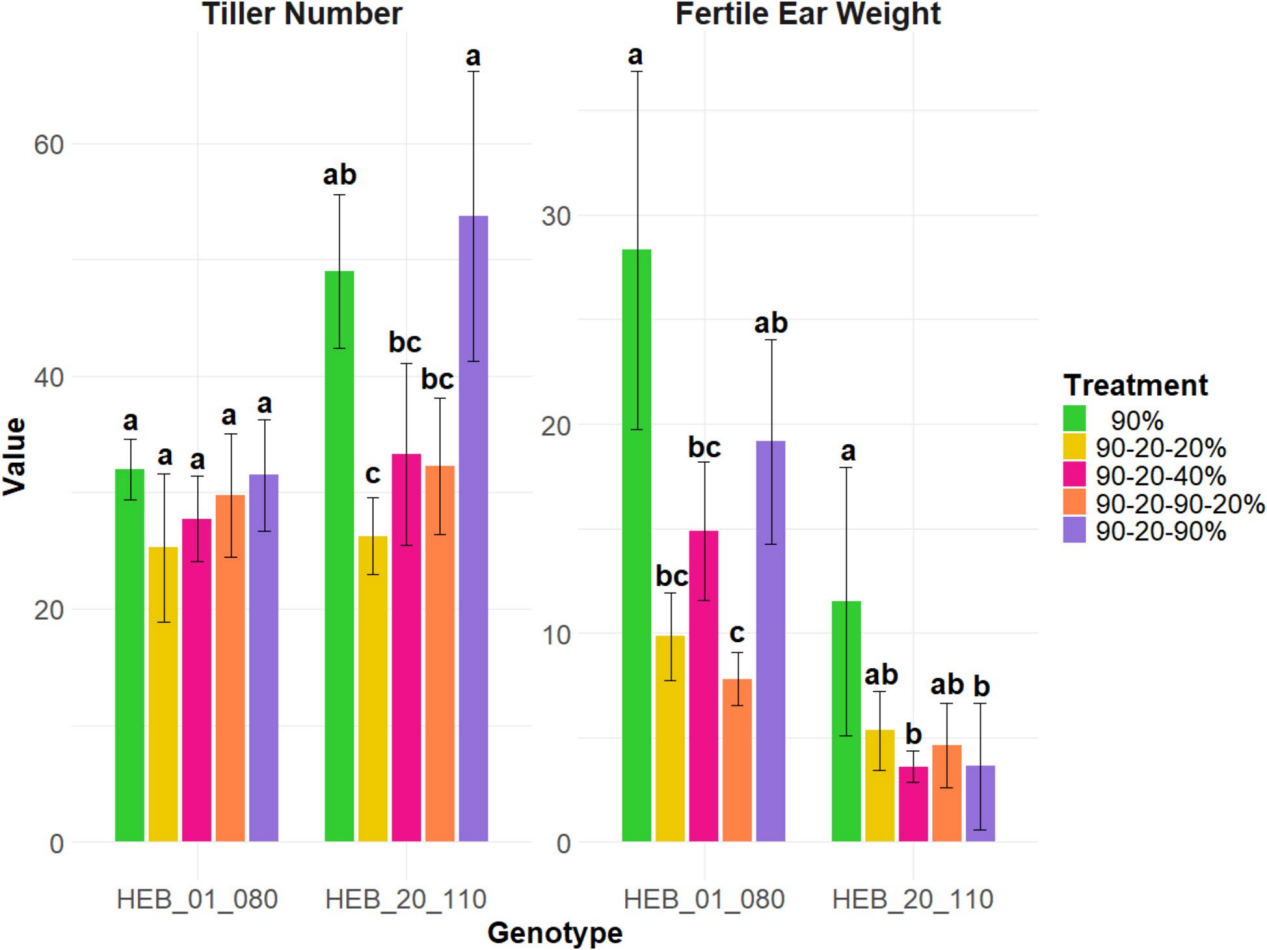
Tiller number at 57 DAS and fertile ear weight for the HEB_01_080 and HEB_20_110 genotypes across five treatments. The treatments are represented by distinct colors. Data are presented as mean ± standard deviation (SD). The statistical analysis was performed using a one-way ANOVA followed by Tukey’s Honest Significant Difference (HSD) test to assess the differences between treatments. Statistically significant differences (P < 0.05) are marked by different letters

**Fig. 15:**
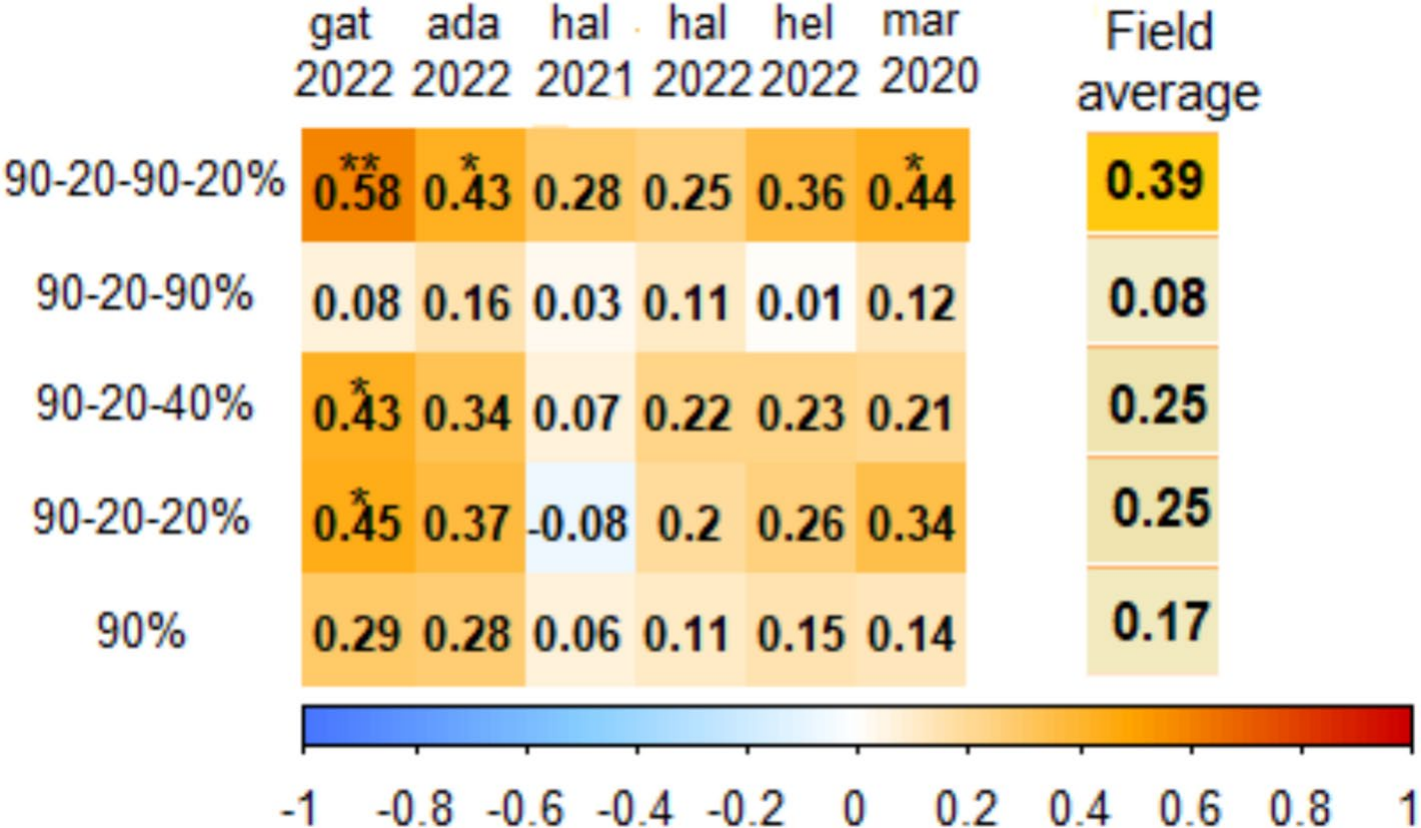
Pearson’s correlation coefficients of fertile ear weight for 26 genotypes under five treatments with grain yield of six different field sites. Significant correlation coefficients are indicated with * *P* < 0.05, **P < 0.01 and *** *P* < 0.001. The 90–20–90–20% treatment showed the highest correlation coefficient in all environments (gat = Gatersleben, Germany; ada = Adana, Turkey; hal = Halle, Germany; hel = Helsinki, Finland; mar = Marchouch, Morocco; [29])

**Fig. 16:**
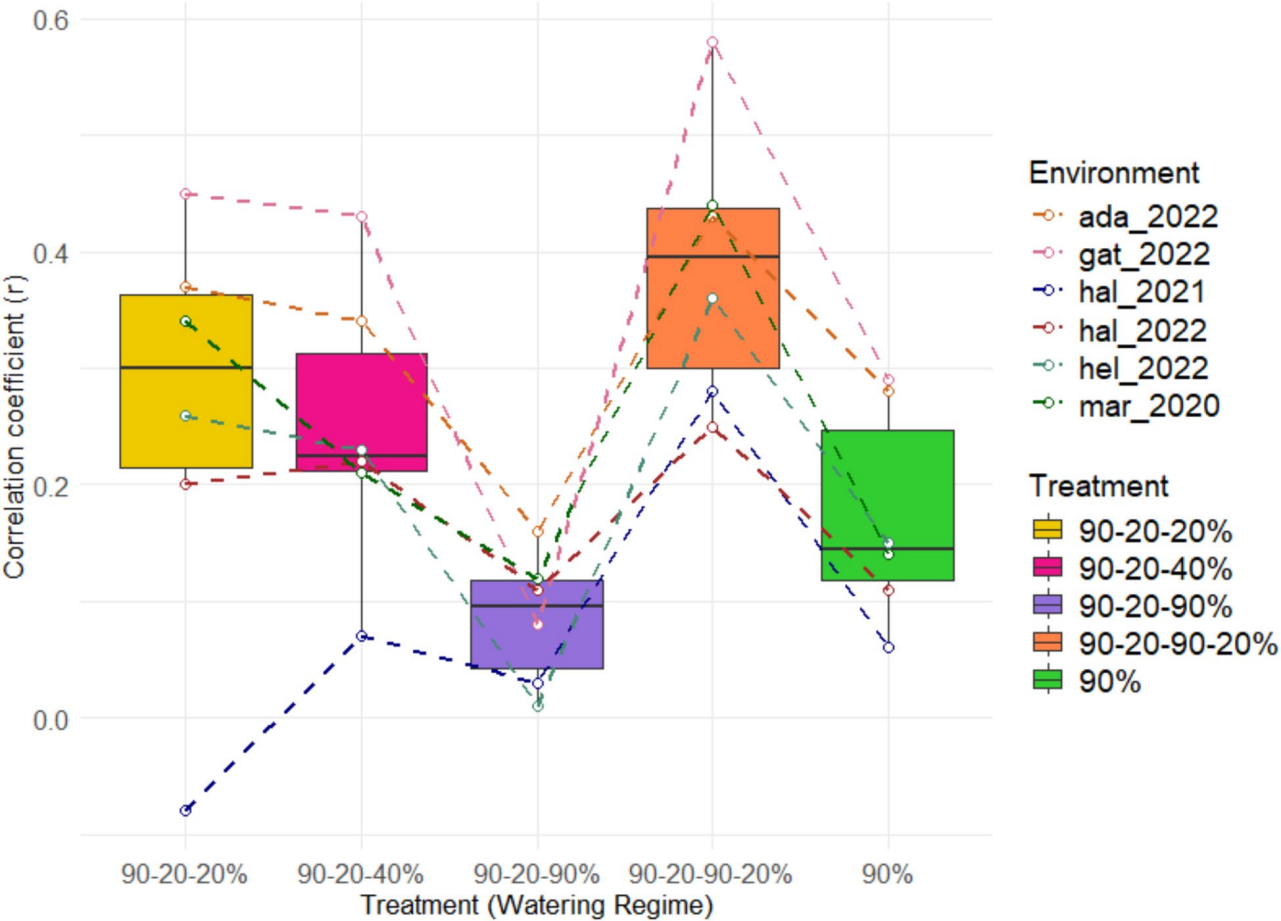
Pearson’s correlation coefficients of fertile ear weight for 26 genotypes under five treatments with grain yield of six different field sites. This graph displays the correlation coefficient (r) of single plant yield Under five treatments with grain yield for six field environments (gat = Gatersleben, Germany; ada = Adana, Turkey; hal = Halle, Germany; hel = Helsinki, Finland; mar = Marchouch, Morocco; [29]), which share 22 genotypes. Each treatment is represented by box plots, which illustrate the distribution of correlation coefficients across the environments. The colored lines connecting the points indicate the trends for each environment across the treatments

Pearson’s correlation coefficients of all traits were calculated per treatment (Fig. 11). Most of the correlations were highly significant. The correlation between tiller number and fertile ear weight (single plant grain yield) was negative for the 90% (control, r = −0.25), 90–20–90% (full recovery, r = −0.20) and 90–20-20% (full stress, r = −0.25) treatments. However, this correlation was not significant in the other treatments, although the general trend remained the same (Fig. 11). The correlation of the slope of tiller number from 90–20-20% (full stress) to 90–20–90% (full recovery) with fertile ear weight and harvest index in the 90–20–90% (full recovery) treatment was also examined (Figs. 12 and 13). The correlation values for both were negative, particularly the correlation between harvest index and the slope of tiller number, which was significantly negative (r = −0.62). This indicates that plants with a higher re-tillering ability were associated with a lower harvest index and fertile ear weight. To this end, HEB_20_110, the highest re-tillering genotype after Barke and HEB_01_080, one of the lowest re-tillering genotypes, were selected for detailed inspection of tiller number and fertile ear weight (Fig. 14). While HEB_20_110 exhibited a consistently high tiller number across all treatments, it showed a lower fertile ear weight under all conditions. These findings further support the general hypothesis of a negative relationship between tiller number and fertile ear weight in these genotypes. Thousand grain weight exhibited a negative correlation with tiller number, but a positive correlation with fertile ear weight across all treatments (Fig. 11). However, the strongest impact on fertile ear weight was observed for grain number across all treatments. Next, we assessed the correlation of fertile ear weight among all genotypes under the various treatments with grain yield data from large plots in previous field experiments conducted across six different environments using the same genotypes [30] (Figs. 15 and 16). Notably, the 90–20–90–20% treatment, simulating field conditions, exhibited the strongest positive correlations in all environments, suggesting it effectively replicated natural conditions for grain yield potential (Figs. 15 and 16). In contrast, both the 90% control treatment and the 90–20–90% full recovery treatment displayed the lowest mean correlations, indicating that the control treatment poorly aligns with actual field data. Additionally, while the full recovery treatment was associated with higher re-tillering, it does not appear to represent a valid experimental setup for practical agricultural implications. Thus, re-tillering may be an effect primarily observed in controlled environments, highlighting the critical role of experimental design in identifying superior genotypes with physiological mechanisms relevant to agriculture.

## Discussion

The number of tillers, or stem branches developing from the plant's base, is usually recorded at different growth stages to assess plant health and productivity. In a study by [32], tiller numbers in barley were recorded at multiple growth stages, including early tillering, mid-tillering, and heading stages. Their study reported that tiller development was significantly controlled by both genetic and environmental factors, with optimal tillering occurring under favorable nutrient and moisture conditions. Similarly, research by [33] highlighted the importance of early tiller formation in wheat, a close relative of barley. They demonstrated that tiller number at the early tillering stage is a reliable indicator of final grain yield, emphasizing the need for precise management practices during this critical period. Our results are consistent with these findings, as we also observed that early tillering was strongly influenced by water availability and genotype, highlighting the importance of both genetic and environmental factors in tiller development. Overall, monitoring the tiller number dynamics may provide valuable insights into crop development and may assist in making informed management decisions to maximize yield potential in barley and other cereals. The Zadoks stage 31 [27] is critical in the phenological development of cereals, where the first detectable elongation of the stem occurs, setting the stage for future yield formation [34]. During early stem elongation, plants are particularly responsive to environmental conditions [35]. The increased sensitivity can magnify the differences in stress tolerance among genotypes, facilitating the identification of resilient varieties [36]. A drought stress occurring at this stage can significantly affect yield components such as tiller number, grain set, and biomass accumulation. By applying stress during the growth period from stem elongation to heading, researchers can study its direct effects on these crucial parameters. These findings suggest that screening genotypes under controlled drought stress at this stage could help to identify varieties with enhanced performance, especially when taking a genotype’s reaction to later water availability into account.

Re-tillering can be a response to environmental conditions, such as water availability, where the plants compensate for stress by generating more tillers to improve their chances of reproductive success. This enhanced re-tillering response indicates a robust recovery mechanism that is activated upon alleviation of water stress, promoting compensatory growth. In our study, we examined the tillering responses of barley genotypes to re-watering. When water availability increased, most genotypes exhibited increased tillering. The treatment in which the genotypes had the highest tiller number was the 90–20–90% (full recovery) treatment. Among the genotypes, HEB_13_142 exhibited the highest tillering, followed by HEB_20_110. In contrast, one of the genotypes that did not exhibit re-tillering under this treatment was the control cultivar Keel. Due to its distinctly different phenology with a very fast development, it might have escaped the drought and switched already to grain filling before re-watering. Several studies have reported a significant negative correlation between tiller number and grain yield under drought stress, indicating that as tiller number increases, the allocation of resources to individual ears and overall yield may decrease [37]. This inverse relationship between vegetative growth (shoot weight) and reproductive yield (ear weight) under stress conditions suggests that resource allocation strategies are critical for optimizing crop performance [38]. We also observed a significant negative correlation between tiller number and fertile ear weight under the 90% (control), 90–20–90% (full recovery), and 90–20-20% (full stress) treatments. Additionally, we found a significant negative correlation between harvest index and re-tillering in the genotypes. These findings suggest that a higher number of tillers does not necessarily result in increased yield or harvest index. Rather, an elevated tiller number may compromise fertile ear weight, suggesting a trade-off between vegetative and reproductive growth under certain environmental conditions. Even without drought stress or other adverse conditions, a trade-off exists where increased tillering can lead to reduced ear weight and yield [39]. These results highlight the complexity of yield formation and the need to consider both tiller number and ear weight when assessing crop productivity.

In agricultural studies, the thousand grain weight is often used as an indicator of grain quality and overall plant health. A higher mean in this metric suggests that the grains produced under this specific treatment were heavier, which could imply better fertility, more favorable growth conditions, or enhanced nutrient availability during critical growth stages. This finding may also indicate that the treatment was effective in optimizing conditions for grain development, leading to improved yield potential. In our study, the negative correlation observed between tiller number and thousand grain weight suggests that increased tillering may lead to a trade-off in resource allocation, thereby reducing grain size due to competition for nutrients and water. Additionally, when tiller number exceeds the plant's potential, sterility of whole tillers can occur, further impacting overall grain yield. Interestingly, grain number exhibited no or even weak negative correlations with tiller number, indicating that excessive tillers do not increase total grain number. Notably, the 90–20–90–20% treatment showed a significantly higher thousand grain weight compared to the control (90%), the 90–20–40% (moderate recovery), and 90–20–90% (full recovery) treatments. Fewer grains were produced under 90–20–90–20% (field conditions simulation) and 90–20-20% (full stress) treatments, allowing for greater assimilate distribution to each grain, probably resulting in a higher starch accumulation per grain, a valuable characteristic of malting barley, which might therefore be suitable to be grown in environments with similar precipitation patterns as in the applied treatments. Taken together, in our study tiller number increases rather caused excess biomass competing for resources than contributing to grain yield.

While the duration was equal, we found that the amount of re-watering following drought also plays a crucial role. A study showed that barley plants subjected to a short drought followed by re-watering produced more fertile ears than those that experienced longer periods of drought without subsequent re-watering [38]. Our observations imply that also the re-watering conditions impact the ratio of sterile ears. It was higher in the 90–20–90% (full recovery), 90–20–90–20% (field conditions simulation) and 90–20–40% (moderate recovery) treatments compared to the 90–20-20% (full stress) and control treatments. Furthermore, the data suggest that an increased amount of water following a period of stress correlates with a higher number of sterile ears.

Finally, we examined the correlation of single plant yield across all genotypes under five treatments with grain yield data of previous field experiments conducted in six environments with the same genotypes [30]. Interestingly, the 90–20–90–20% (field conditions simulation) treatment exhibited the highest positive correlation at each of the six field environments, followed by the 90–20-20% treatment. As those two treatments generally showed the lowest tiller numbers, we assume that light interception, which is generally higher in single plant pot experiments than in field conditions, might have driven this relationship. Tiller number in the remaining treatments, might in contrast have led to an overestimation of tiller number that is not directly comparable to field conditions. The 90–20–90–20% (field conditions simulation) treatment, thus, produced results more congruent with diverse field conditions compared to the other stress treatments tested, and may be optimal to simulate natural field conditions regarding grain yield potential.

## Conclusions

Drought is one of the most important factors restricting agricultural production, which seriously affects crop yield [40, 41]. Recoverability in plants like barley upon drought stress is crucial as it determines their ability to regain growth, maintain yield, and ensure survival in fluctuating environments. This trait allows barley to exploit available resources more efficiently following stress periods, ultimately enhancing its adaptability to climate variability.

Having a greater amount of water after drought stress can lead to an increase in the number of ears in barley due to improved photosynthesis, enhanced carbohydrate uptake and better hormonal growth regulation. Adequate re-watering facilitates nutrient absorption and energy production, which are critical for reproductive development. However, if re-watering occurs excessively, it may not sufficiently support the transition from tillers to fertile ears, resulting in sterility.

Our study enabled non-invasive phenotyping of the plant architecture and biomass production under high-throughput conditions under automated watering according to different implemented drought and re-watering scenarios. Ultimately, this contributed to a better understanding of the re-tillering capacity, a rarely investigated trait and proposed potential directions for future barley breeding.

A greater amount of water after stress, such as in the 90–20–90% (full recovery) treatment, increases the tiller number more than in the 90% (control) treatment. However, the key point is that a high tiller number does not necessarily correlate with a high number of fertile ears. In fact, fertile ear weight showed a significantly negative correlation with tiller number across the 90% (control), 90–20-20% (full stress), and 90–20–90% (full recovery) treatments. This suggests that while the plants respond to re-watering by producing more tillers, the physiological cost of generating additional tillers could be a reduction in the resources available for grain filling, leading to smaller and/or fewer fertile ears. In general, we observed the trend that a low re-tillering behavior of genotypes is linked with higher grain yield, as genotypes with high yield in field trials, including the highly plastic check cultivar RGT Planet, showed only weak re-tillering. Apparently, some HEB lines might harbor promising alleles to increase the harvest index under re-watering scenarios, as they clearly outperformed their reference parent Barke in our experiments. Identifying these beneficial alleles by means of genome-wide association studies could be the scope of future experiments.

Based on correlating our stress data with field data we found that the control treatment is poorly linked to true field data. Also the full recovery treatment, in our study associated with the highest re-tillering, does not seem to represent an experimental setup allowing for implications for practical agriculture. That said, re-tillering as an explanatory factor for grain yield expression after drought stress seems to be a rather unique phenomenon of studies conducted in controlled environments. Although the correlation of re-tillering and grain yield was obvious, the practical implications for breeding are limited as tiller counting is a laborious and weakly heritable trait. However, due to the high grain yield correlations of field data with the 90–20–90–20% treatment, this strategy could be adopted to screen for grain yield in early breeding generations. These findings underscore the importance of planning a realistic design in controlled environment experiments in order to improve the identification of stress-resilient genotypes selected for subsequent cultivation in field trials and, potentially, in practical agriculture.

## Supplementary Information

Additional file 1. Selection of the genotypes used in this study

Additional file 2. Image of the phenotyping facility with 520 pots

Additional file 3. Summary of the trial setup showing the sequence of steps

Additional file 4. Continuation strategy of watering regimes after transfer from phenotyping facility

Additional file 5. Visual differences between treatments regarding plant height and shoot density

Additional file 6. Complete raw phenotype data of the study

## Data Availability

The dataset supporting the conclusions of this article is included within the article (Additional file 6).
