# Supplementary figures and images for: Tillering plasticity of drought-stressed barley genotypes under different re-watering regimes
Source: BMC Plant Biol. 2025 Oct 13;25:1368. doi: 10.1186/s12870-025-07504-8 (PMC12516877; doi:10.1186/s12870-025-07504-8)

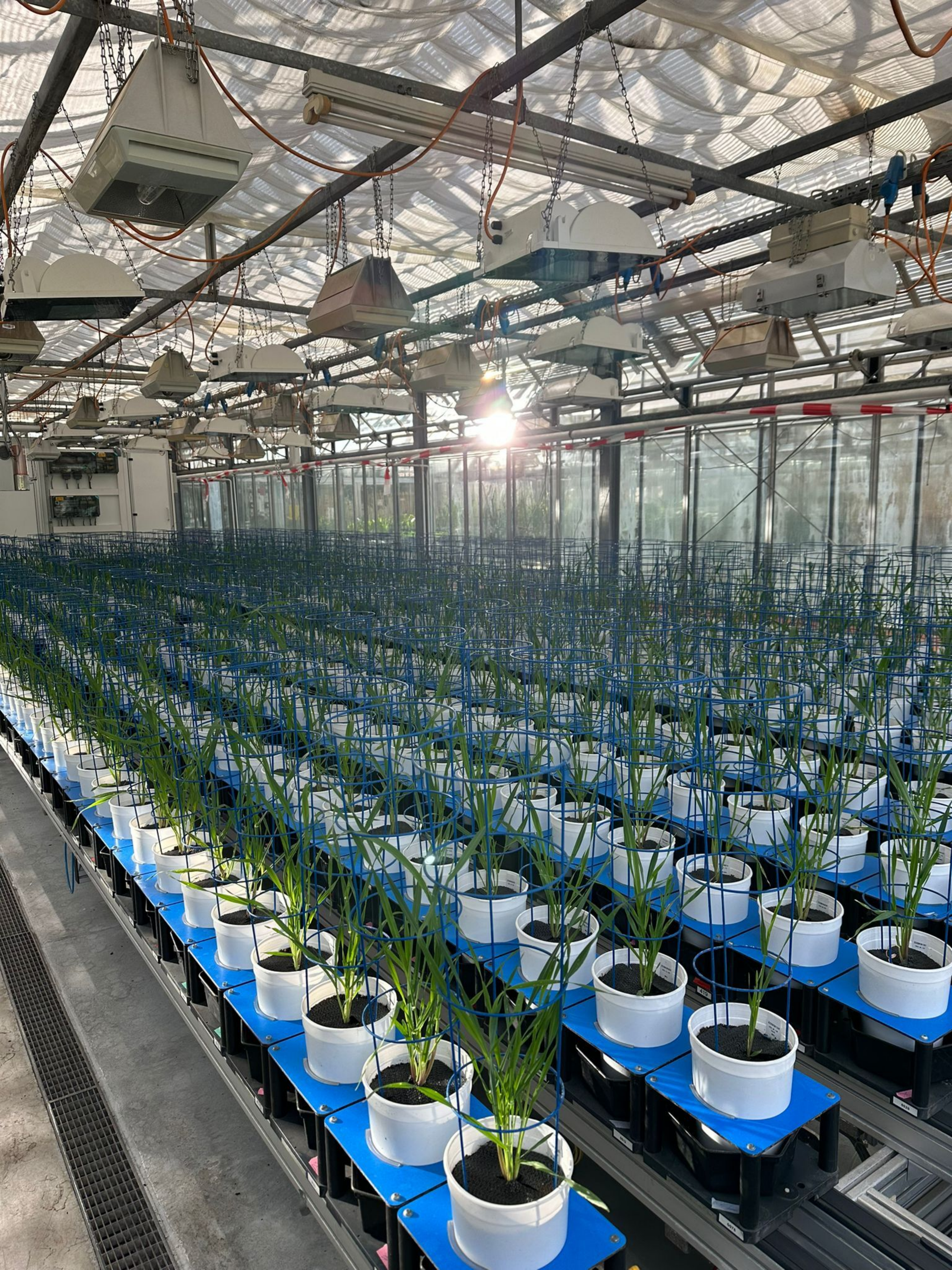

Supplement: Supplementary file 2 — Additional file 2. Image of the phenotyping facility with 520 pots [file 12870_2025_7504_MOESM2_ESM.pdf]

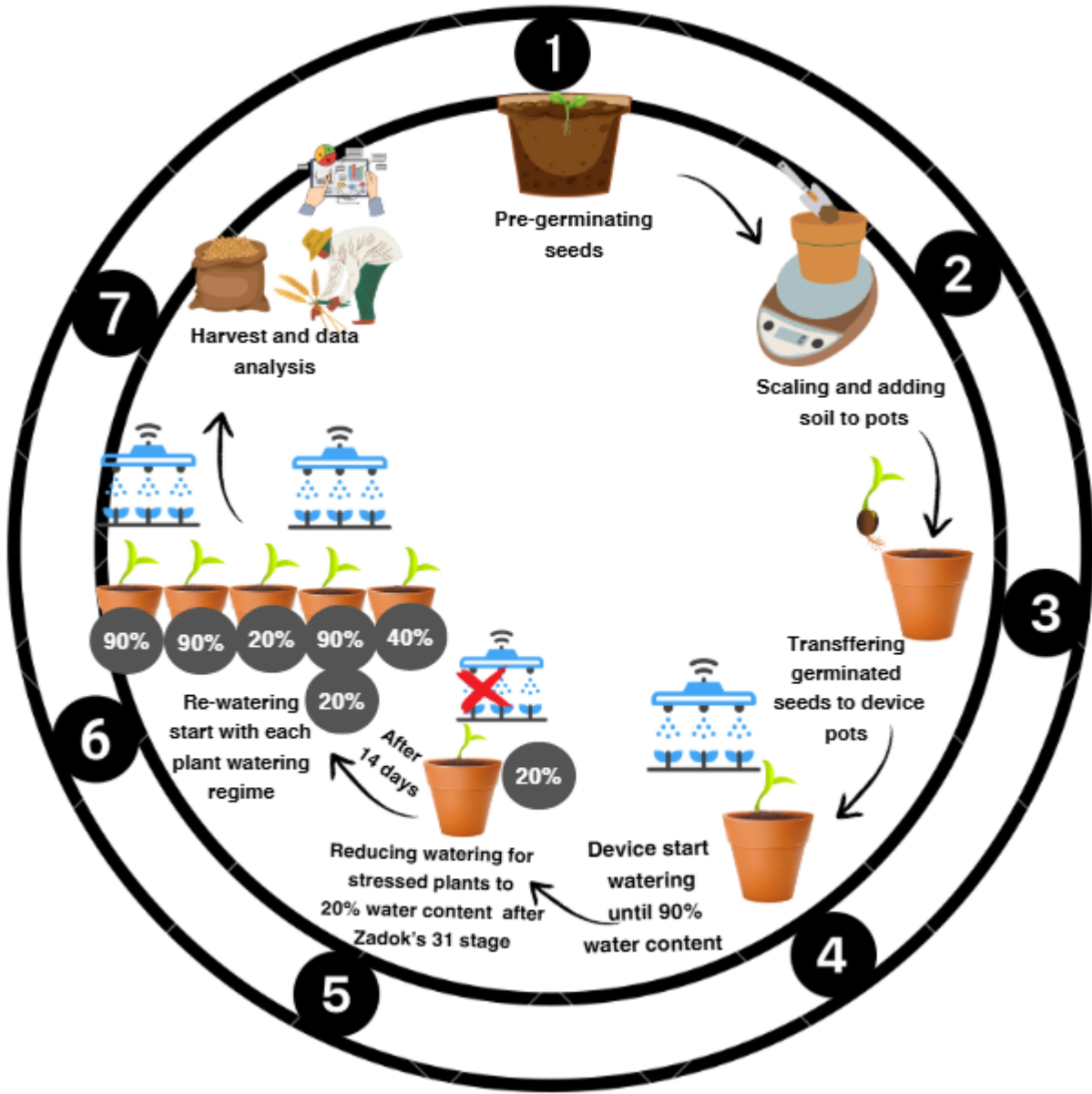

Supplement: Supplementary file 3 — Additional file 3. Summary of the trial setup showing the sequence of steps [file 12870_2025_7504_MOESM3_ESM.pdf]

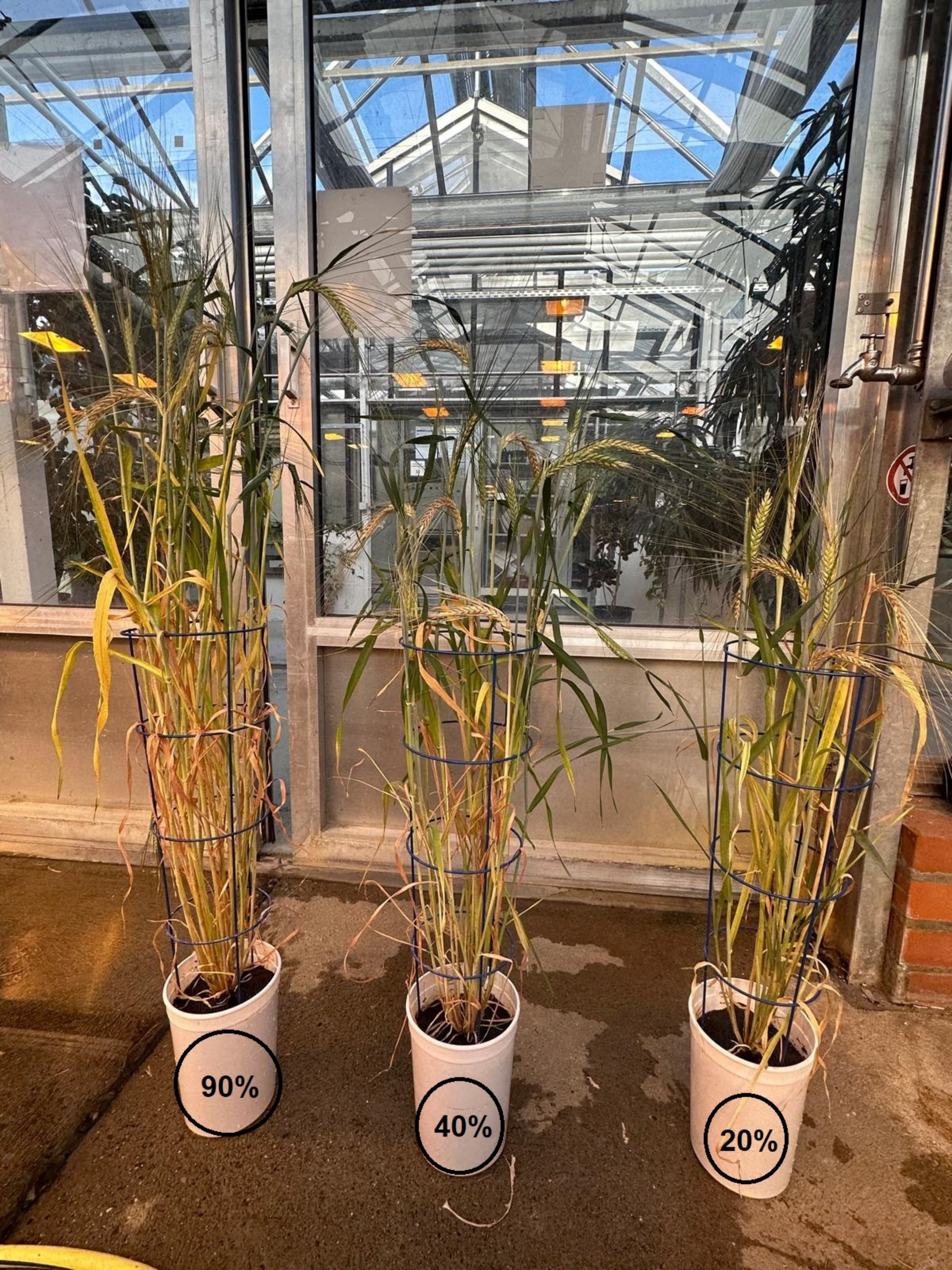

90%

40%

20%

Supplement: Supplementary file 5 — Additional file 5. Visual differences between treatments regarding plant height and shoot density [file 12870_2025_7504_MOESM5_ESM.pdf]
